# Supplementary material for: Quantitative proteomics and single-nucleus transcriptomics of the sinus node elucidates the foundation of cardiac pacemaking
Source: Nat Commun. 2019 Jun 28;10:2889. doi: 10.1038/s41467-019-10709-9 (PMC6599035; doi:10.1038/s41467-019-10709-9)
Supplement: Supplementary file 1 — Supplementary Information [file 41467_2019_10709_MOESM1_ESM.pdf]

Quantitative proteomics and single-nucleus transcriptomics of the sinus node elucidates the foundation of cardiac pacemaking

Linscheid *et al.*

SUPPLEMENTARY DISCUSSION

More about the membrane clock and ion channels of the sinus node

The Na^+ channel, $Na_v1.5$/Scn5a, was detected at similar levels in the sinus node and atrial muscle (Figure 2b). In the rabbit[1], $Na_v1.5$/Scn5a and the corresponding Na^+ current, I_{Na}, are absent from the centre of the sinus node, but present in the periphery of the sinus node (but at a reduced level compared to atrial muscle). In the mouse sinus node, there is a similar distribution, but I_{Na} is reported to be more prominent than in other species[2]. It is likely that the tissue biopsies used in the present study included both the centre and periphery of the sinus node. At the transcript level (again based on tissue biopsies), $Na_v1.5$/Scn5a has previously been reported to be similarly expressed in the sinus node and atrial muscle in the mouse[3]. Therefore, the finding that $Na_v1.5$/Scn5a was similar in the sinus node and atrial muscle may be a shortcoming of the sampling technique. Mutations in Scn5a are responsible for hereditary sinus node disease[2], consistent with a direct or indirect role for $Na_v1.5$/Scn5a in pacemaking. K^+ channels were equally expressed in the sinus node and atrial muscle or were more highly expressed in the atrial muscle. ERG/$K_v11.1$/Kcnh2 and K_vLQT1/$K_v7.1$/Kcnq1 are two important voltage-gated K^+ channels, responsible for $I_{K,r}$ and $I_{K,s}$ respectively[4]. $I_{K,r}$ and $I_{K,s}$ are not thought to be important in the mouse heart[5]. However, ERG/$K_v11.1$/Kcnh2 and K_vLQT1/$K_v7.1$/Kcnq1 were relatively abundant in the sinus node and atrial muscle (Figure 2 and Figure 4). ERG and K_vLQT1 mRNA is also reported to be highly abundant in the rat sinus node[6]. This suggests that the role of voltage-gated K^+ channels in the rodent heart needs to be revised. There is discussion about which subunits are responsible for the ATP-sensitive K^+ channel responsible for $I_{K,ATP}$[5]. $K_{ir}6.2$/Kcnj11 and SUR2/Abcc9 are most likely responsible[5]. In the mouse sinus node and atrial muscle, $K_{ir}6.2$/Kcnj11 was the most abundant $K_{ir}6$ isoform, although expression of $K_{ir}6.1$/Kcnj8 was also substantial ($K_{ir}6.2$/Kcnj11:$K_{ir}6.1$/Kcnj8) (Figure 2 and Figure 4). Of the accessory SUR subunits, only SUR2/Abcc9 was detected (Figure 4). It is, therefore, likely that these channel subunits are responsible for $I_{K,ATP}$.

Missing ion channels

Although ~50 ion channel subunits were detected in this study, several ion channel subunits expected to be expressed in the tissue were not detected. We were expecting to detect $Ca_v1.3$/Cacna1d and $Ca_v3.1$/Cacna1g, because they have been shown to play an important role in the moue sinus node[7]. No definitive evidence for the presence of the two Ca^{2+} channels was obtained. It is possible that the channels were present, but could not be discriminated from $Ca_v1.2$/Cacna1c and $Ca_v3.2$/Cacna1h (detected sequences were common to both channels in each case). We did not detect $K_{ir}2.1$/Kcnj2 or any other $K_{ir}2$ channel in any sample. $K_{ir}2.1$/Kcnj2 and related $K_{ir}2$ isoforms are thought to be responsible for the background inward rectifier K^+ current, $I_{K,1}$, and the generation of the resting potential in the atrial and ventricular muscle[8]. $K_{ir}2$ channel subunits may have been undetectable because of a technical issue (although other K_{ir} channel subunits were detected), or simply due to the stochastic nature of the sampling technique. However, their absence from our dataset raises the possibility that $K_{ir}2$ channels are not responsible for $I_{K,1}$ in the mouse atria. It is well known that the density of $K_{ir}2$ channels and $I_{K,1}$ is lower in the atria than in the ventricles and the inward rectification displayed by $I_{K,1}$ is weaker in the atria[9]; in the dog, the abundance of $K_{ir}2.1$ is 76.9-fold higher and the density of $I_{K,1}$ is ~10-fold higher in the ventricles than the atria[9]. Is it possible that $K_{ir}2$ channels are not responsible for $I_{K,1}$ in the atria? It is also well known that the density of $I_{K,1}$ is lower in the centre of the sinus node than in the atrial muscle (to facilitate pacemaking)[10]. Several other K^+ channels show an appropriate expression pattern: both $K_{ir}3.1$/Kcnj3 and TASK-1/Kcnk3 were expressed at a high level in the atrial muscle and a low level in the sinus node (Figure 2 and Figure 4) consistent with what is known of $I_{K,1}$. Together with $K_{ir}3.4$/Kcnj5, $K_{ir}3.1$/Kcnj3 forms the inward rectifying ACh-activated K^+ channel responsible for $I_{K,ACh}$. There is evidence of a constitutively active $K_{ir}3$-based inward rectifier K^+ channel in the heart[11] and this could be a substitute for $K_{ir}2.1$/Kcnj2. $K_{ir}3$ channels are known to contribute to the resting potential in neurons[12]. Another interesting alternative could be the twin pore K^+ channel, TASK-1/Kcnk3. At positive potentials, it has a weak outward rectifying characteristic and, at more negative potentials, the current-voltage relationship is either linear or possibly weakly inward rectifying[13, 14]. TASK-1 is responsible for the resting potential in pulmonary artery smooth muscle cells[15]. Other ion channel subunits that were expected, but were not detected, are $K_v1.4$, $K_v1.5$, $K_v4.2$, $K_v4.3$ and KChIP2. Once again it is possible that they were simply not measured due to the stochastic nature of the sampling, but it is also possible that these ion channel subunits are absent or of very low

abundance. In this case, reported membrane currents would have to be explained by alternative channels (as in the case of $I_{K,1}$). Our data showed evidence for possible expression of Na_v1.6, Na_v1.7, Na_v1.8, Ca_v1.1, Ca_v1.4, Ca_vβ4 and K_{ir}3.2. However, due to large parts of identical amino acid sequences with other identified channels, these channels could not be distinguished with certainty.

Stoichiometric relationship between sarcolemmal and intracellular Ca^{2+}-handling proteins

In skeletal muscle with voltage-dependent sarcoplasmic reticulum Ca^{2+} release and thus tight coupling between the Ca^{2+} channel and Ca^{2+} release, the Ryr:Ca^{2+} channel ratio is ~0.5, whereas, in ventricle with Ca^{2+}-dependent sarcoplasmic reticulum Ca^{2+} release and thus less tight coupling, the ratio varies from 3.7-10.2 in different mammals[16]. In the mouse sinus node, we calculate a ratio of 17 from the mass spectrometry data. This suggests less tight coupling than in the ventricle; this is to be expected because there are no t-tubules in the sinus node. In the mouse sinus node, the ratio of Serca2:Ryr2 is 400:1 (based on ion channel copy numbers); both Serca2 and Ryr2 will be generating the same Ca^{2+} fluxes (at steady-state) and it is interesting that the ATP-driven Ca^{2+} pump (Serca2) is much less efficient than the channel (Ryr2). It is also interesting that the abundances of potential sarcoplasmic reticulum ion channels (TricA/Tmem38a, TricB/Tmem38b, Mg23/Tmem109 and Clic[17]) that compensate for the charge carried by Ca^{2+} as it exits through Ryr2 are comparable to that of Ryr2 (Supplementary Figure 7).

Kinases

One of the important kinases regulating the activities of the Ca^{2+} clock proteins is the Ca^{2+}/calmodulin-dependent protein kinase type II (Camk2). Among the Camk2 subunits, we found CamkIIδ/Camk2d to be the most abundant, and furthermore it was significantly more highly expressed in the atrial muscle. CamkIIγ/Camk2g also showed a tendency for higher expression in the atrial muscle, while Camk2α/Camk2a and Camk2β/Camk2b showed a tendency towards higher expression in the sinus node, albeit none of these differences were statistically significant. Two related kinases, Camk1 and Camk1d showed a tendency for higher expression in the atrial muscle. The kinases, cAMP-dependent protein kinase (PKA) and 5-AMP-activated protein kinase (AMPK), were also quantified in both tissues and some of their subunits showed a tendency for higher expression in the atrial muscle.

Contractile proteins

Contractile proteins are known to vary regionally in the heart: for example, myofilaments are known to be differentially expressed between the atria and ventricles[18-21]. It is thought that sinus node cells have a paucity of contractile proteins and the sinus node can contain 'empty' cells devoid of contractile proteins[22]. However, our dataset shows that mouse sinus node myocytes do contain lots of contractile proteins. Our data revealed that myofilaments are differentially expressed between the sinus node and atrial muscle. Of the myosin heavy chains, we find that Myh6 is the most abundant in the mouse tissues and Myh4, Myh6, Myh7, Myh10 and Myh11 have surprisingly higher expression in the sinus node than in atrial muscle. For the myofilament light chains, Myl3, Myl4 and Myl7 are the most abundant forms in mouse tissues, where Myl1, Myl4 and Myl7 have higher expression levels in the sinus node compared to atrial muscle. Myosin heavy chain forms the actomyosin cross-bridge. Myh6 (α-myosin heavy chain) is associated with higher actomyosin ATPase activity compared with Myh7 (β-myosin heavy chain), which is the other cardiac myosin heavy chain isoform[23]. The fact that Myh6 is the most abundant myosin heavy chain in the dataset is expected, because Myh6 is considered to be the atrial isoform – Myh7 is considered to be the ventricular isoform. In the adult human heart, the ratio of Myh6:Myh7 is 9 in non-failing atria and 0.05 in non-failing ventricles[23]. In the present study, the ratio of Myh6:Myh7 was 900 in the atrial muscle and 200 in the sinus node, i.e. Myh6 is the predominant isoform, but the proportion of Myh7 is greater in the sinus node. Wessels et al.[24] reported that, in the neonatal human heart, whereas Myh6 is expressed in the atrial muscle and sinus node, Myh7 is expressed in the sinoatrial junction. The greater proportion of Myh7 in the sinus node in the present study is consistent with this. Surprisingly, genome-wide association studies have demonstrated an association between Myh6 and sinus node function in the general population[25]. Furthermore, Myh6 mutations have been linked to familial sick sinus syndrome[25]. Myosin light chain forms another part of the actomyosin cross-bridge, providing structural support for the neck region of the myosin heavy chain and modulating actomyosin ATPase activity[26]. In the human, Myl4 and Myl7 are atrial isoforms,

whereas Myl3 is the ventricular isoform[26]. In the present study, Myl3, Myl4 and Myl7 were therefore surprisingly of equal abundance in the sinus node. However, Myl4 and Myl7 as expected had higher expression levels in the sinus node as compared to atrial muscle. The skeletal muscle isoform, Myl1, was surprisingly abundant in the sinus node and more abundant than in the atrial muscle. Again surprisingly, familial bradyarrhythmias requiring pacemaker implantation (as well as atrial cardiomyopathy) have been linked to mutations in Myl4[26]. This is the first study to quantify important contractile proteins in the sinus node linked to sinus node dysfunction.

Natriuretic peptide system and exocytosis

Natriuretic peptides are powerful and important regulators of the cardiovascular system known to be secreted by the atria. Natriuretic peptides are known not to be secreted by the sinus node: Liu et al.[27] have previously shown that HCN4 and Nppa (atrial natriuretic peptide) expression exclude each other in the mouse sinus node: whereas HCN4 is highly expressed, Nppa has very low expression in the sinus node. Our study shows that not only Nppa itself, but also closely connected components of the natriuretic peptide system (Nppb, Npr3, Tbx5, Corin and others; Supplementary Figure 13) are expressed at lower levels in the sinus node than atrial muscle. Interestingly, Nppb (brain natriuretic peptide) was even more differentially expressed between tissues than the previously known atrial muscle marker, Nppa. Amongst many actions on the cardiovascular system, natriuretic peptides affect the electrophysiology of the sinus node and atrial muscle[28]. Therefore, it is not surprising that in this study the three natriuretic peptide receptors, Npr1-3, were detected in both tissues (Supplementary Figure 13). In the sinus node, the most abundant receptor is Npr3, although it was significantly more highly expressed in the atrial muscle (Supplementary Figure 13). Npr3 knockout mice have sinus node dysfunction and increased susceptibility to atrial fibrillation[28]. Natriuretic peptides are secreted from atrial myocytes by exocytosis and Figure 7 shows that proteins associated with exocytosis are more highly expressed in atrial muscle as are Nppa and Nppb; it is possible the two are connected.

Transcription factors

Transcription factors are responsible for the formation and function of the sinus node. A total of 78 transcription factors were detected, although some could not be quantified. Of these, 19 showed a significant difference between the sinus node and atrial muscle (Supplementary Figure 21). Over the last decade, much has been learnt about the transcription factors that drive the embryonic development of the sinus node[29]. The surprising finding is that few of the transcription factors involved in the development of the sinus node were detected in the adult sinus node. Transcription factors, Tbx5, Shox2, Isl1 and Tbx3, drive the sinus node gene programme and Tbx3 and Tbx18 repress the atrial muscle gene programme[29]. Pitx2 prevents development of a left-sided sinus node and Nkx2-5, Tbx20 and, curiously, Tbx5 drive the atrial muscle gene programme[29]. Bmp4 is another key regulator of sinus node programming[29]. Of these only Tbx5, Tbx20 and Nkx2-5 were measurable; Tbx3 was detected in one sinus node sample. Why many transcription factors known to be responsible for the embryonic development of the sinus node were not detected is not known – however, a likely possibility is that they are not required in adult tissue with fully differentiated cells. Tbx5 and Tbx20 were significantly less abundant in the sinus node than the atrial muscle; Nkx2-5 tended to be distributed in a similar manner (Supplementary Figure 21); as drivers of the atrial muscle gene programme, it is to be expected that they should be more abundant in the atrial muscle. The role of the other transcription factors showing a significant difference between the sinus node and atrial muscle (Supplementary Figure 21) is not known and the identification of their roles is potentially important.

Lipid metabolism

The metabolic profile of the sinus node is heavily associated with the synthesis, accumulation and storage of lipids. Differences were found in numerous metabolic pathways associated with lipid, glucose and protein metabolism (Figure 7) but the proteins with the greatest sinus node expression were mainly associated with lipid synthesis, storage, and lipogenic pathways such as the thyroid hormone receptor pathway (fatty acid synthase, FASN; Acetoacetyl-CoA synthetase, Aacs; Diacylglycerol O-acyltransferase 1, Dgat1; Perilipin-1, Plin1; Resistin, Retn; Acetyl-CoA carboxylase 1, Acaca; ATP-citrate synthase, Acly; Thyroid hormone-inducible hepatic protein, Thrsp)[30]. Furthermore, PPARγ is an upstream regulator of the

overexpressed proteins; this pathway regulates adipocyte differentiation, lipid uptake and adipogenesis[31]. Several proteins involved with lipid synthesis and storage that were overexpressed in the sinus node are either routinely expressed in cardiomyocytes (Gpd1, Acaca, Dgat1 and Acly; Figure 6A)[32, 33] or upregulated (Fasn, Scd1) under certain conditions, such as exercise training[34] or disease[35-37]. Collectively, these results suggest that storage may be more common in the sinus node than in atrial muscle. This is significant because fatty acids are the major source of energy for the heart. Given that many of the lipogenic proteins are predominantly or exclusively associated with adipocytes, one interpretation of our results is that the sinus node is surrounded by cardiac adipose tissue. The finding of overexpression of UCP1 (~23-fold) in the sinus node supports this contention, as cardiac adipose tissue is known to express thermogenic genes uniquely associated with brown adipose tissue. Furthermore, histological analyses of the sinus node have shown adipocytes surround and infiltrate the sinus node[38]. Epicardial adipose tissue (EAT) accounts for ~20% of total heart weight and covers 80% of cardiac surfaces[39]. Lipid storage is particularly important for the heart, because fatty acids are the primary fuel source for cardiomyocytes; therefore, the proximity of EAT allows for rapid lipid mobilisation which supports the high energy demands of cardiomyocytes. In addition to energy storage, the close proximity of EAT has led to the suggestion that adipocytes directly modulate cardiomyocyte function[40]. Several physiological roles have been suggested, including regulation of vascular tone, protection of cardiomyocytes from lipid toxicity and regulation of redox state[41]. In addition, the presence of thermogenic proteins (UCP1) in EAT suggests adipose tissue provides direct heat to the myocardium and the coronary vasculature[42, 43]. Nevertheless, while adipose tissue is clearly important for normal cardiac function, and "browning" of adipocytes may even be cardioprotective, excessive adipose deposition is associated with a range of cardiac diseases including atrial fibrillation, coronary artery disease and heart failure[44].

The sinus node sample was also enriched with proteins involved in lipid droplet formation. While most of these proteins are adipose-specific (Plin1, Lipe and Retn), one of them (Dgat1) is also found in cardiomyocytes, suggesting lipid droplets may be more common in sinus node cells than atrial myocytes[45]. Intracellular stores of lipid are usually low in cardiomyocytes as fatty acids are predominantly obtained extracellularly from the circulation[46]. Nevertheless, under certain conditions, lipid droplets may contribute significantly to mitochondrial fatty acid supply. In addition, lipid droplets are now known to participate in a wide range of cellular processes, including modulation of protein availability, fatty acid trafficking, lipid signalling and viral defences[47]. Furthermore, numerous studies have shown lipid droplets protect against lipid toxicity by accumulating bioactive lipids when fatty acid concentrations become too high[48]. However, similar to excess cardiac adipose tissue, the accumulation of lipid droplets is associated with disease, including heart failure and dilated cardiomyopathy[49]. In this respect, the sinus node may be particularly vulnerable to lipotoxicity in a disease setting.

In addition to lipid storage, proteins associated with ketolysis and ketongenesis (Acat2, Bdh1, Acly, Aacs) were overexpressed in the sinus node, compared to the atria. Ketone bodies are predominantly produced from fatty acids in the liver and subsequently transported to extrahepatic tissues for utilisation[50]. Once synthesised and released into the circulation, ketone bodies can be catabolised for ATP generation, converted in to lipids, or excreted in the urine. While oxidation of fatty acids has the highest theoretical yield of ATP per carbon unit, ketone body oxidation is more energetically favourable. Therefore, ketone bodies act as an alternative fuel source during a variety of physiological states, including: periods of fasting, starvation, neonatal development and pregnancy[50]. In addition to an alternative energy source and sink, ketone bodies play important roles in cellular signalling and transcription[51], and may have therapeutic implications for cardiovascular disease[52]. Therefore, the overrepresentation of proteins associated with ketone bodies in the sinus node may have ramifications on a wide range of cellular pathways.

Limitations of the study

The study we present here was conducted on mice. Available evidence suggests that study of the sinus node of laboratory animals (e.g. mouse, rat, rabbit) helps in understanding the human sinus node. Pacemaker ionic currents and the pacemaker transcriptome of the mouse and human sinus nodes are similar and animal models of many different conditions (pregnancy, postnatal development, ageing, from

day to night, athletic training, heart failure, myocardial infarction, metabolic syndrome, diabetes) replicate the same sinus node phenotype as observed in humans[10, 53-55]. For the atrial proteomes we have the possibility to compare the similarities between the murine right atrial proteome we measured here with human right atrial proteomes that were recently published[32]. Mapping human Uniprot identifiers to mouse Uniprot identifiers through BioMart enabled us to find a human ortholog in the human right atrial proteome for more than 80% of the mouse right atrial proteins we quantified in this study. Evaluating the protein abundances in murine right atria to protein abundances in human right atria present a correlation of 0.73. This indicates that the right atrial proteomes of the two species resemble each other. This is an important observation from a translational point of view. Yet, it is important to stress that the data presented in this study are based on mice and care must be taken to assess the suitability of mouse models for each given research question individually.

Furthermore, our work is based on analysis of biopsy samples. Although this is the standard in fields measuring cardiac proteomes or transcriptomes, it does represent a challenge. Work on biopsies suffer the general limitation that tissue is composed of different cell types, as we also explicitly show in our study. Cell sorting of acutely dissociated cells offers a potential solution to this problem. However, whereas millions of ventricular or atrial myocytes can be isolated from a heart, from the mouse heart we can isolate ~300 sinus node cells. With current state-of-the-art technology it is not feasible to measure proteomes of a deep and quantitative quality based on so few cells. Another possibility would be to use a sinus node-like cell line, but it is not known how faithful such cell lines are and a study of the proteome of a cell line would be premature. Conversely, our study of the proteome of the natural sinus node will be a reference point for any sinus node cell line.

In this study, we did not use a molecular reporter to identify the sinus node and take a biopsy, contrary to what others have done[56]. Instead the site of biopsy was guided by our previous studies of the anatomy of the sinus node using histology or micro-CT, immunolabeling of marker proteins such as HCN4 to confirm the nature of the tissue, mapping the electrical activity of multicellular sinus node preparations (for example using extracellular potential recording) to identify the leading pacemaker site, and recording of intracellular action potentials from in and around the leading pacemaker site with sharp microelectrodes[27]. Based on our years of experience from these studies, we know where the leading pacemaker site is located in the mouse heart and we took a biopsy at this location: at the bifurcation of the sinus node artery in the intercaval region towards the superior vena cava.

SUPPLEMENTARY TABLES

Supplementary Table 1: List of primary and secondary antibodies used for immunohistochemistry.

Target protein	Primary antibody (dilution; catalogue no., supplier)	Secondary antibody (dilution; catalogue no., supplier)
HCN4	rabbit polyclonal anti-HCN4 (1:100; APC-052, Alomone Labs, Israel)	donkey anti-rabbit Fluorescein conjugate (1:100; AP182F, Millipore, USA) or donkey anti-rabbit Cy3 conjugate (1:400; AP182C, Millipore, USA)
Cx43	rabbit polyclonal anti-Cx43 (1:1000; C6219, Sigma-Aldrich, UK)	donkey anti-rabbit, Fluorescein conjugate (1:100; AP182F, Millipore, USA)
TASK-1	rabbit polyclonal anti-KCNK3 (1:10; APC-024, Alomone Labs, Israel)	donkey anti-rabbit Cy3 conjugate (1:400; AP182C, Millipore, USA)
MAGP-1	goat polyclonal anti-MAGP-1 (1:200; sc-166075, Santa Cruz Biotechnology, USA)	donkey anti-goat Alexa Fluor 546 (1:400; A11056, Thermo Fisher Scientific, UK)
Laminin	rabbit polyclonal anti-laminin (1:200; L9393, Sigma-Aldrich, UK),	donkey anti-rabbit Cy3 conjugate (1:400; AP182C, Millipore, USA)
Elastin	rabbit polyclonal anti-elastin (1:100; ab21610, abcam, UK),	donkey anti-rabbit Cy3 conjugate (1:400; AP182C, Millipore, USA)
Collagen IV	rabbit polyclonal anti-collagen type IV (1:100; AB756P, Merck, UK)	goat anti-rabbit Alexa Fluor 488 (1:400; A11034, Thermo Fisher Scientific, UK)

Supplementary Table 2: Ion channel protein expression data used for action potential modelling. Expression in the sinus node is shown as a ratio of expression in the atrial muscle.

			**Label-free quantification**	**Normalised raw intensities**
**Gene name**	**Protein name**	**Physiological process**	**Mean±SEM**	**Mean±SEM**
Hcn1	HCN1	$I_{f,HCN1}$	4±1.1	13±5
Hcn4	HCN4	$I_{f,HCN4}$	526±99	151±38
Scn5a	Nav1.5	I_{Na}	1.4±0.1	1.2±0.1
Cacna1c	Cav1.2	$I_{Ca,L}$	0.9±0.1	0.8±0.1
Cacna1h	Cav3.2	$I_{Ca,T}$	1.5±0.2	1.5±0.5
Kcnh2	ERG	$I_{K,r}$	1.3±0.2	2.0±0.1
Kcnj3	Kir3.1	$I_{K,ACh}$	0.7±0.1	0.6±0.1
Kcnk3	TASK1	Not known	0.8±0.03	
Atp2a2	SERCA2	Sarcoplasmic reticulum Ca^{2+} uptake	1.0±0.1	0.8±0.1
Ryr2	RYR2	Sarcoplasmic reticulm Ca^{2+} release	0.9±0.0	0.8±0.0

Supplementary Table 3: Action potential characteristics of the atrial model and converted sinus node-like model. dV/dt_{max}, maximum upstroke velocity; APD_{50}/APD_{90}, action potential duration at 50% or 90% repolarization.

	**Atrial muscle**		**Sinus node**	
	**Label-free quantification**	**Normalised raw intensities**	**Label-free quantification**	**Normalised raw intensities**
**Maximum diastolic potential (mV)**	-76.5	-77.9	-75.9	-76.3
**Overshoot (mV)**	16.4	17.9	2.4	2.3
**dV/dt_{max} (mV/ms)**	118.4	130.1	14.2	14.8
**APD_{50} (ms)**	5.4	5.4	19.2	17.4
**APD_{90} (ms)**	24.3	25.1	33.1	29.8
**Cycle length (ms)**	-	-	191	180
**Diastolic deparization rate (mV/ms)**	0	0	0.15	0.16

Supplementary Table 4: Single channel conductances used for Markov chain ion channel models.

**Gene**	**Protein**	**Ionic current**	**Single channel conductance (pS)**
Hcn1	HCN1	$I_{f,HCN1}$	0.98
Hcn4	HCN4	$I_{f,HCN4}$	0.98
Cacna1c	$Ca_v1.2$	$I_{Ca,L}$	6.9
Cacna1h	$Ca_v3.2$	$I_{Ca,T}$	4.7
Kcnh2	ERG	$I_{K,r}$	1.6

SUPPLEMENTARY FIGURES

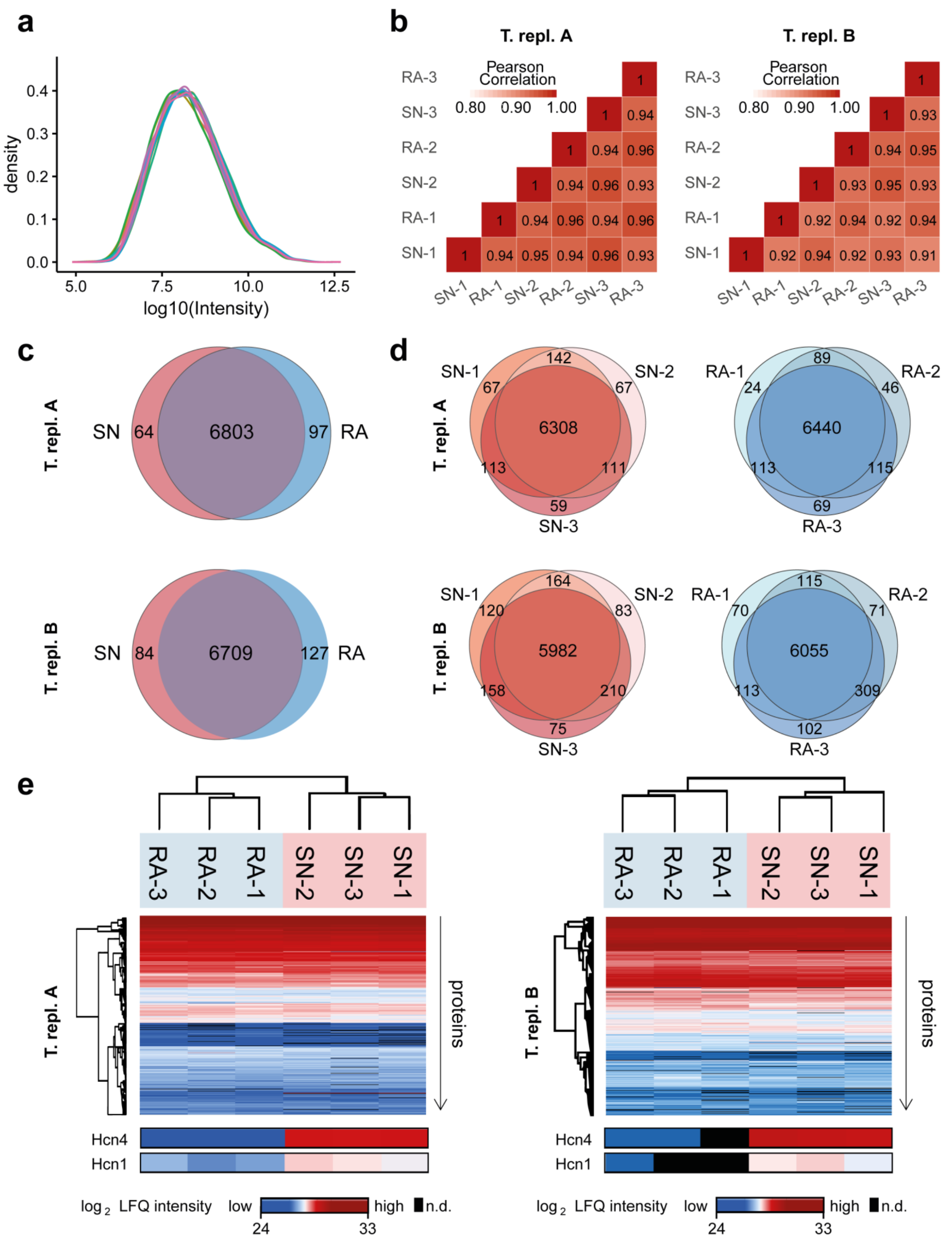

Supplementary Figure 1: High-resolution proteomics measurement of sinus node and adjacent atrial muscle. a. Histograms of sample raw intensity distributions show high conformity, indicating minimal technical variation **b.** Correlation matrix for proteome samples show high correlation between samples in both technical replicates **c.** Venn diagrams showing overlap in protein identifications between tissues. The majority of proteins were identified in both sinus node and atrial muscle in both technical replicates, enabling quantitative comparison of proteins across the cardiac regions. **d.** Venn diagrams of biological replicates show that most proteins were identified in all three biological replicates, indicating availability of repeated measurements for most proteins in the dataset. Technical replicate B yielded slightly fewer protein identifications overall due to quality of LC-MS/MS runs. **e.** Unsupervised hierarchical clustering shows grouping of sinus node and atrial muscle samples into distinct clusters reproducibly in both technical replicates. LFQ, label-free quantification; RA, right atrial muscle; SN, sinus node; SN-1 etc., biological replicates; T. repl. A/T. repl. B, technical replicates.

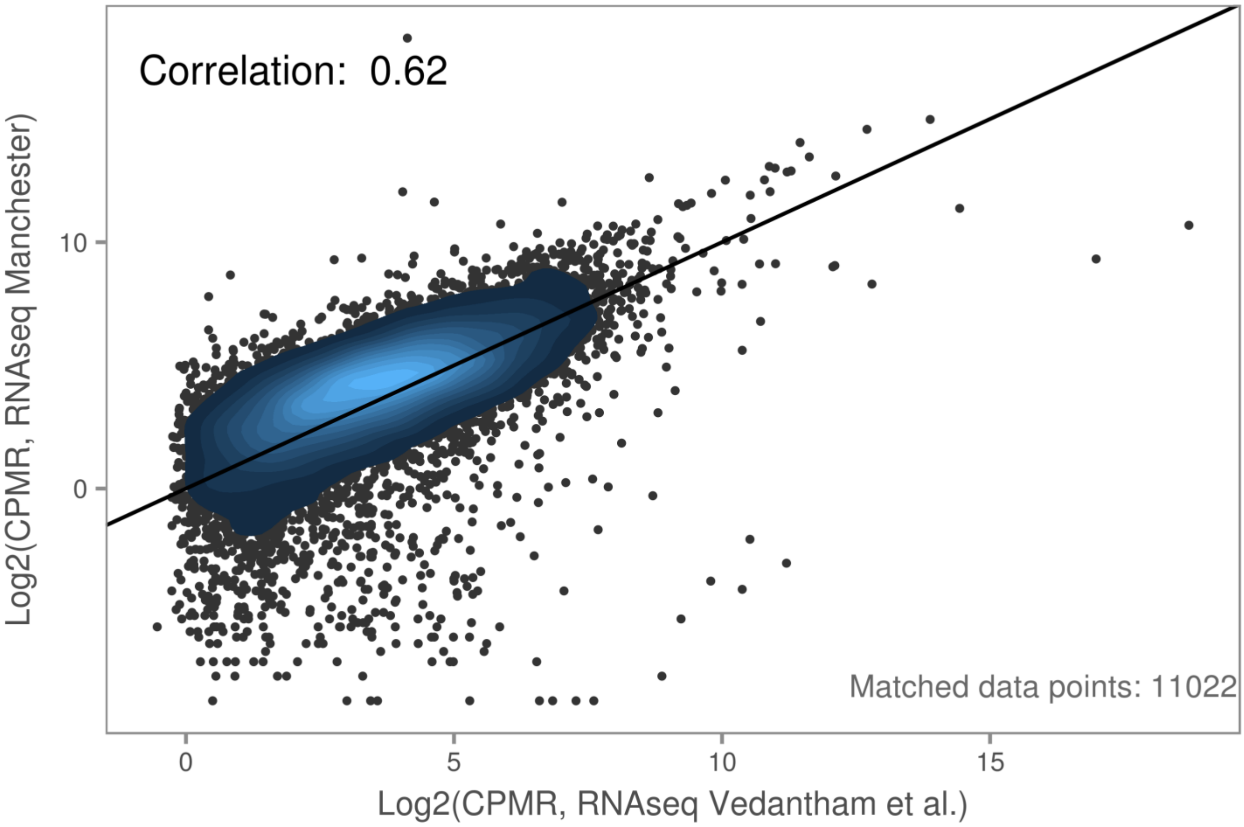

Supplementary Figure 2: Comparison of mRNA sequencing results in sinus node form laser microdissection versus tissue biopsy protocol. Two-dimensional density distribution of mRNA sequencing data from Vedantham *et al.*[56] obtained from laser microdissection of a reporter mouse strain (x axis) versus mRNA sequencing data of our tissue isolation protocol (y axis) shows correlation of 0.62 and centering around identity line (light-blue line; light blue part of distributions contains highest point density, black part the lowest point density). Analysis based on 11022 matching data points between datasets. CPMR: counts per million reads.

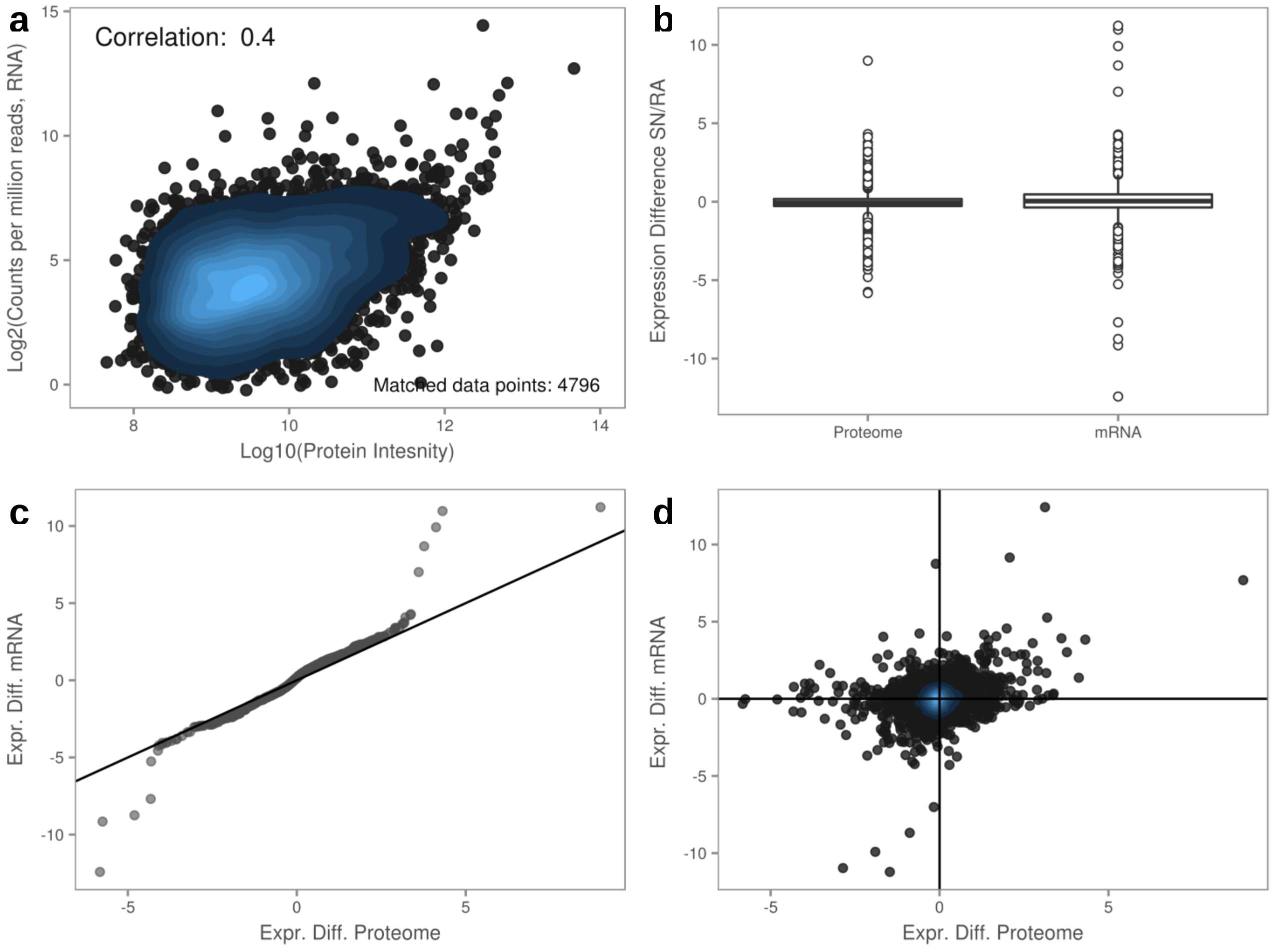

Supplementary Figure 3: Comparison of SN vs RA expression differences in mRNA and proteome data. a. Correlation of protein intensity from our study to mRNA counts from Vedantham et al. Color gradient reflects point density (light blue = highest point density). **b.** Boxplots of difference distributions of proteome and mRNA data show wider spread of mRNA distribution, but similarity in central quantiles. Boxplots represent median and interquartile range, whiskers represent 5th-95th percentiles. Outliers are represented as empty circles **c.** Quantile-quantile plot comparing both distributions shows divergence mainly in the most extreme values, while central part of the distributions are similar. **d.** Two-dimensional density distribution of mRNA and protein rations shows centering of the distribution at zero (light blue = highest point density, black = lowest point density). “Expr. Diff.” always indicates expression difference of SN / RA.

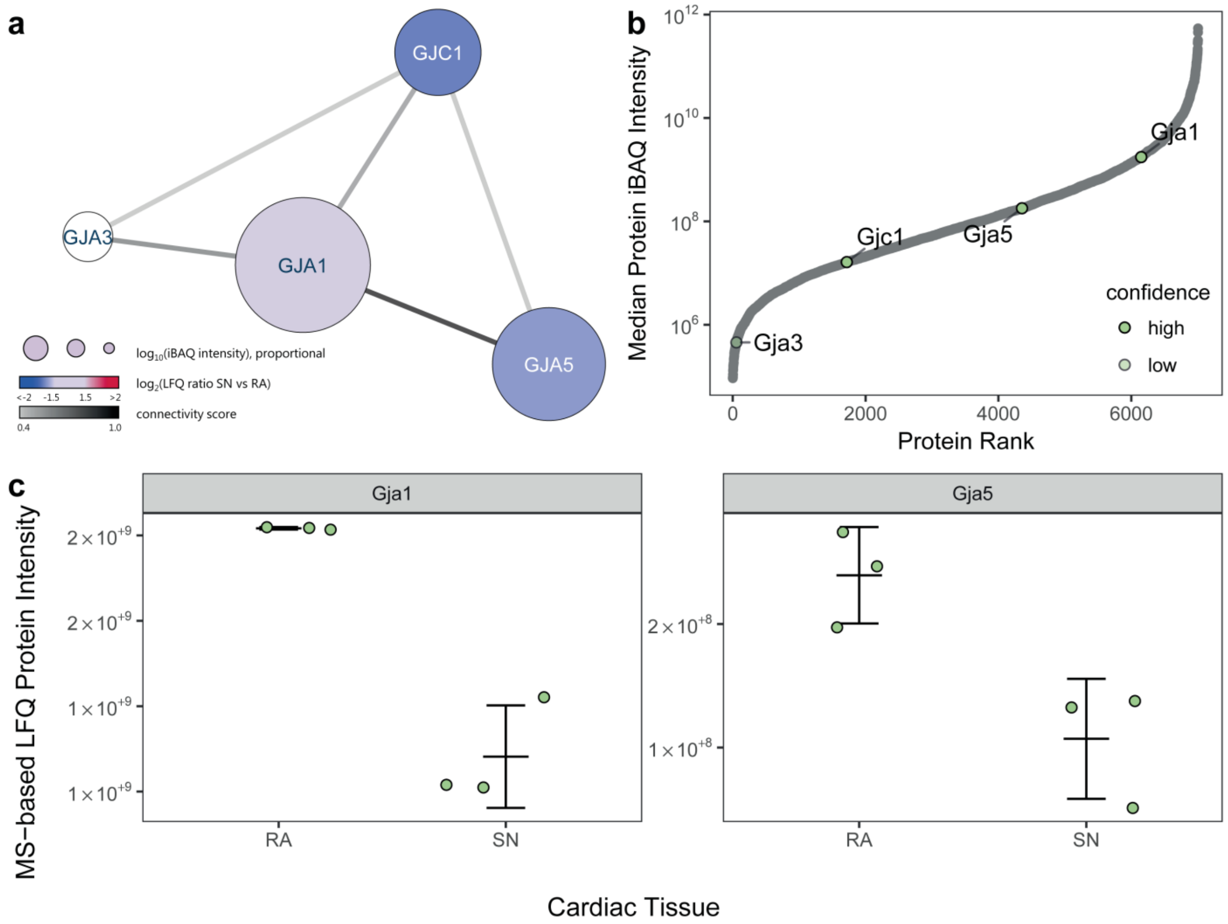

Supplementary Figure 4: Protein expression of connexins in sinus node and atrial muscle. a. Protein association networks retrieved from STRING[57] shows differential expression of proteins between sinus node and atrial muscle. Nodes are coloured by significance (red, significantly higher abundance in sinus node; blue, significantly higher abundance in atrial muscle) and node size represents relative protein abundance. **b.** Rank plot showing all proteins in the dataset ranked from lowest to highest intensity. Proteins of interest are highlighted. **c.** Protein expression in sinus node and atrial muscle for significantly differentially expressed proteins in the network (Student's t-test p-value<0.05). iBAQ, intensity-based absolute quantification; LFQ, label-free quantification; RA, right atrial muscle; SN, sinus node. Error bars represent mean ± standard deviation. . Source data are provided as a Source Data file.

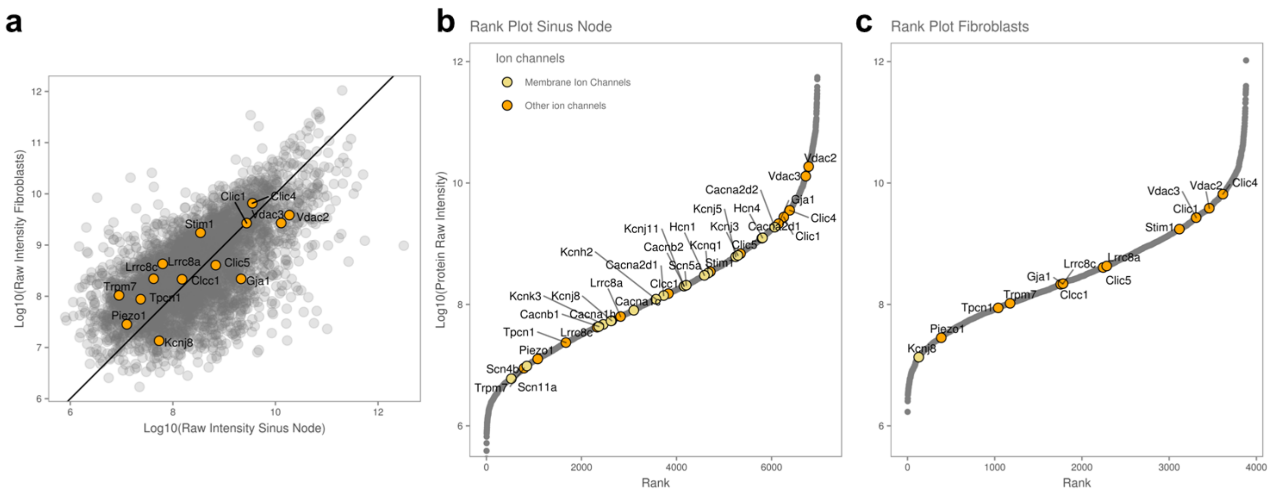

Supplementary Figure 5: Comparison of SN tissue proteome with proteome of isolated cardiac fibroblasts. a. Correlation between protein intensities in both datasets was r=0.63. All ion channels identified in both datasets are highlighted. **b.+c.** Rank plots of protein identifications for both datasets. Ion channels are highlighted as identified in each of the datasets, showing that most membrane ion channels (yellow) were not identified in fibroblasts.

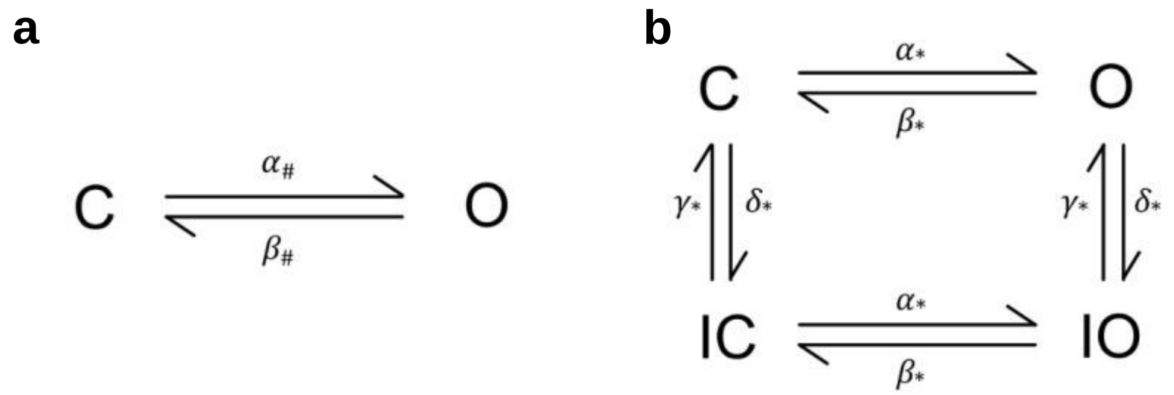

Supplementary Figure 6: Markov chain models used in simulation. a. Closed and open state model used for HCN1 and HCN4. **b.** Model with closed, open and two inactive states used for Cav1.2, Cav3.2 and ERG.

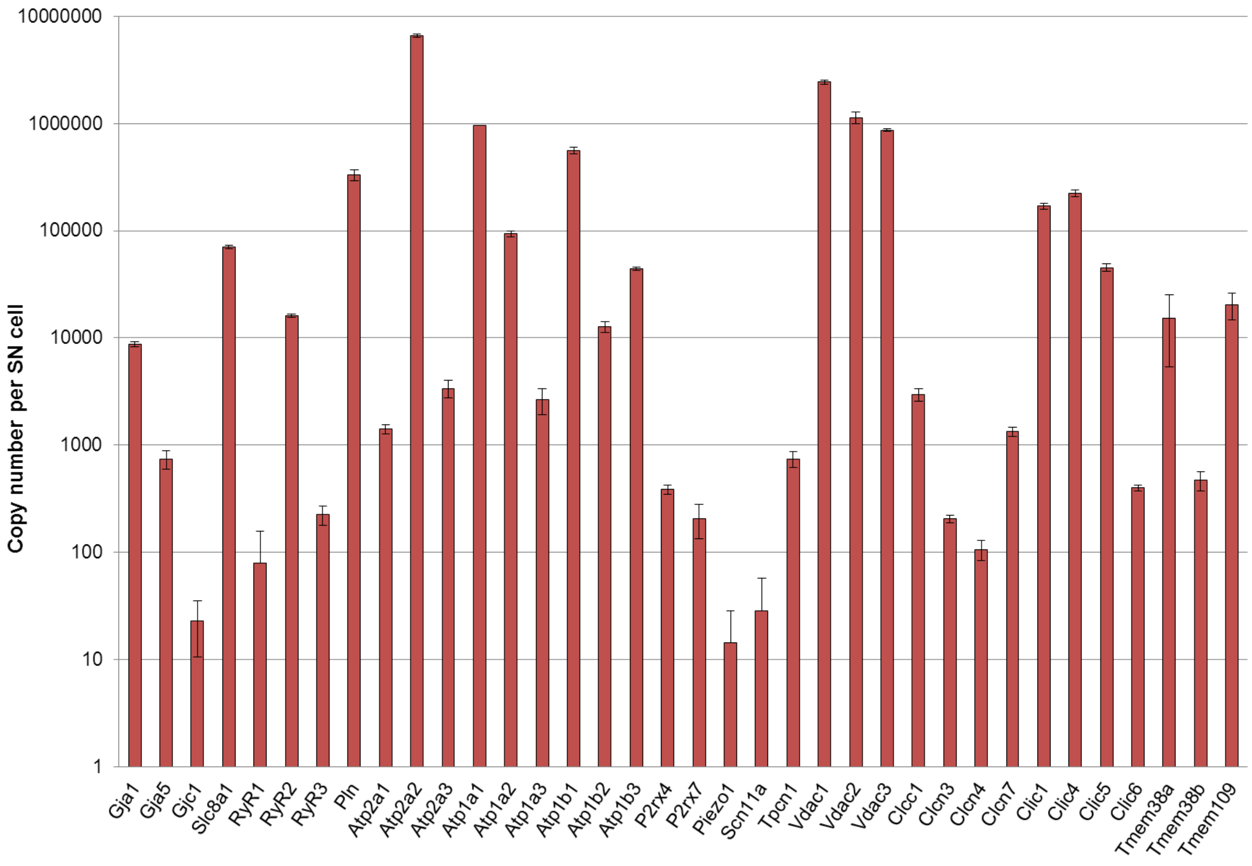

Supplementary Figure 7: Copy numbers per cell calculated for sinus node for proteins involved in ion transport and conduction. Means ± standard error of mean are shown. Note that copy numbers are displayed on a logarithmic scale. See Supplementary Data 3 for details of the calculation.

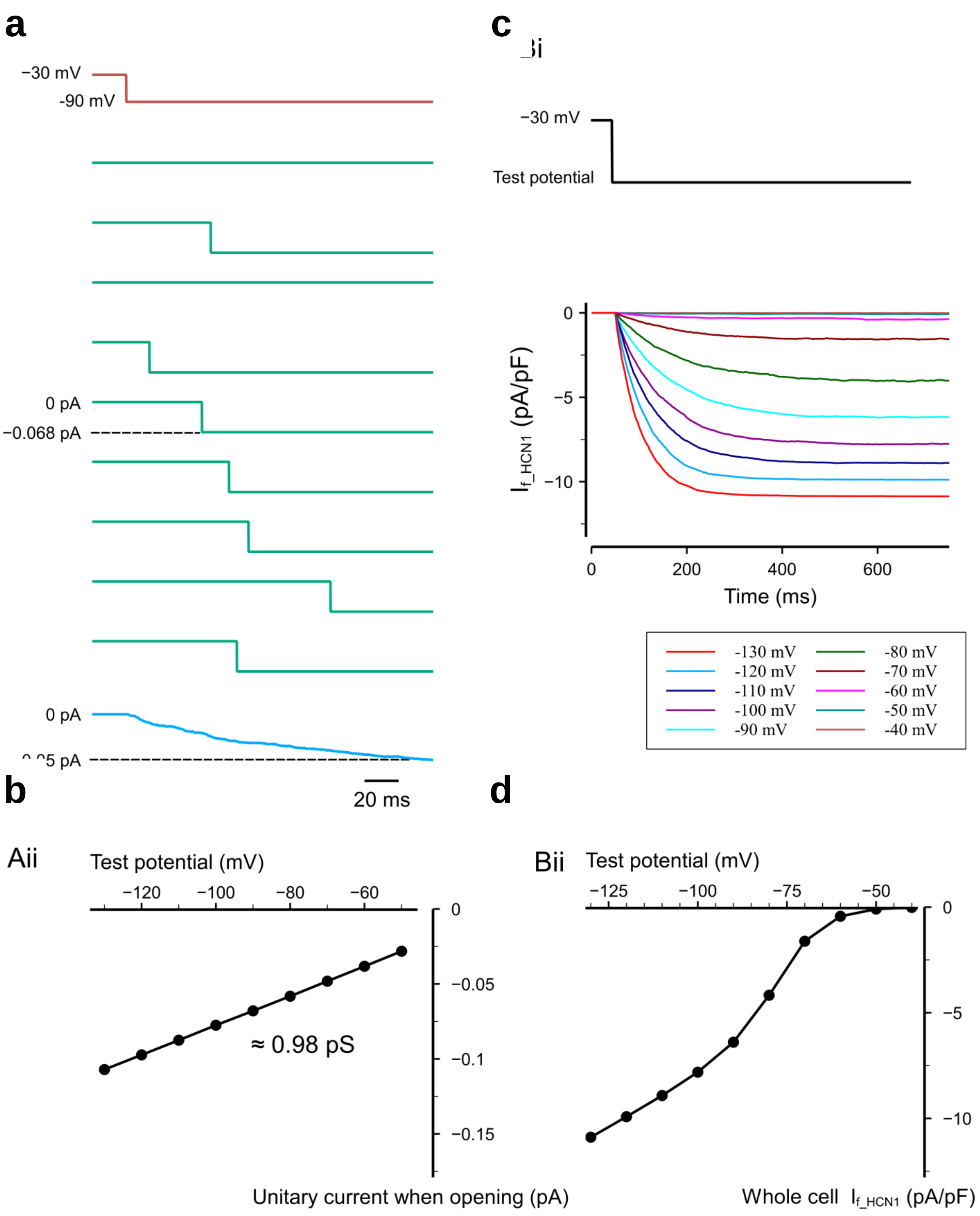

Supplementary Figure 8: Unitary and whole cell currents through the Hcn1 channel. a. Voltage clamp protocol (red), unitary current traces (green; 9 consecutive sweeps shown) and ensemble average current from 200 consecutive sweeps (blue). The channel was held at -30 mV for 500 ms and then tested at -90 mV for 200 ms. **b.** Current-voltage relationship of the unitary HCN1 channel. Solid circles show the maximum current when the channel was open during 10 sweeps. The slope of the line indicating the unitary channel conductance was 0.98 pS. **c.** Whole cell current from 2,550 unitary Hcn1 channels with a holding potential of -30 mV and test potentials from -130 mV to -40 mV. **d.** Current-voltage relationship of the whole cell Hcn1 current.

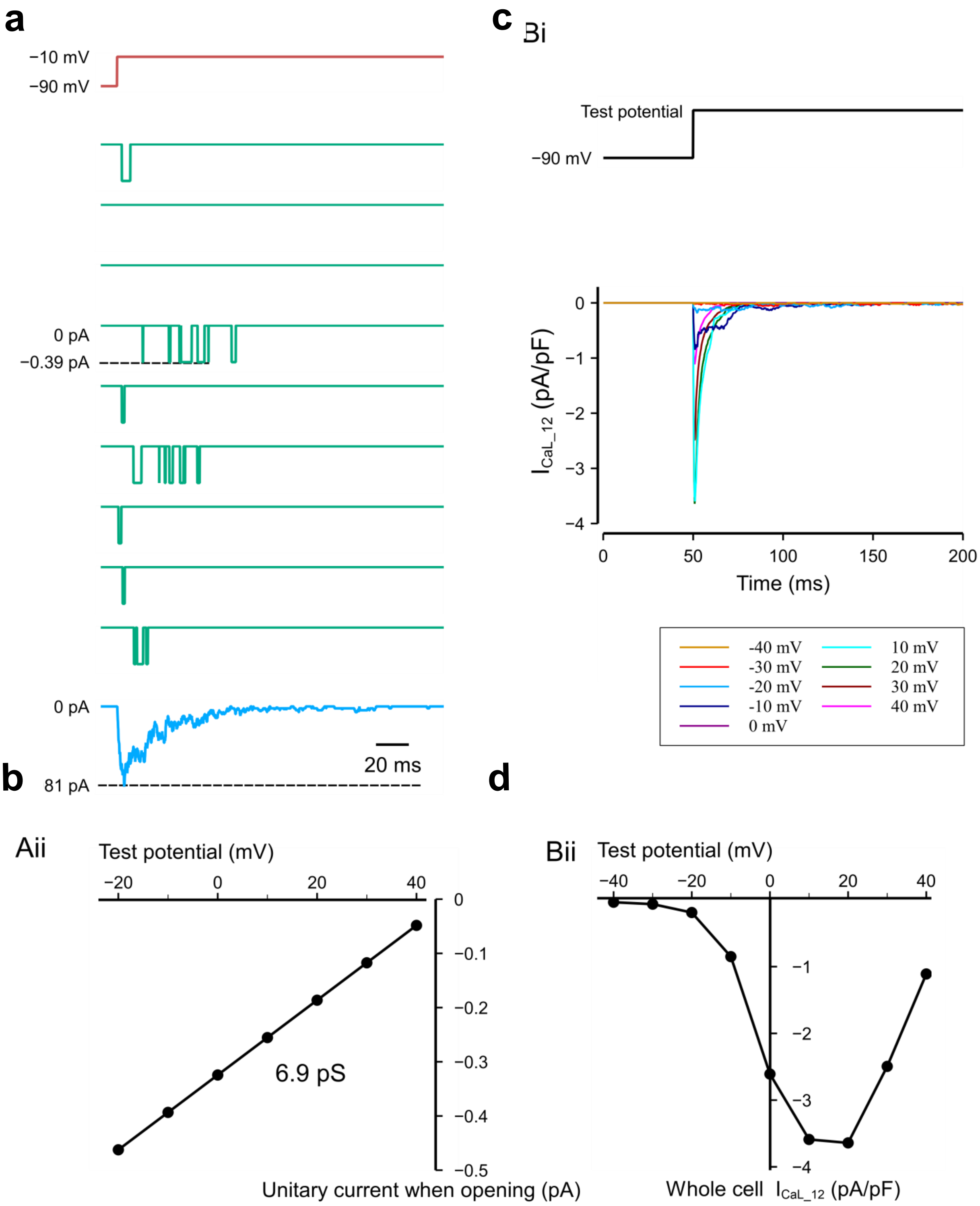

Supplementary Figure 9: Unitary and whole cell currents through the L-type Ca^{2+} channel ($Ca_v1.2$). a. Voltage clamp protocol (red), unitary current traces (green; 9 consecutive sweeps shown) and ensemble average current from 200 consecutive sweeps (blue). The channel was held at -90 mV for 500 ms and then tested at -10 mV for 200 ms. **b.** Current-voltage relationship of the unitary $Ca_v1.2$ channel. Solid circles show the maximum current when the channel was open during 10 sweeps. The slope of the line indicating the unitary channel conductance was 6.9 pS. **c.** Whole cell current from 870 unitary channels with a holding potential of -90 mV and test potentials from -40 mV to 40 mV. **d.** Current-voltage relationship of the whole cell $Ca_v1.2$ current.

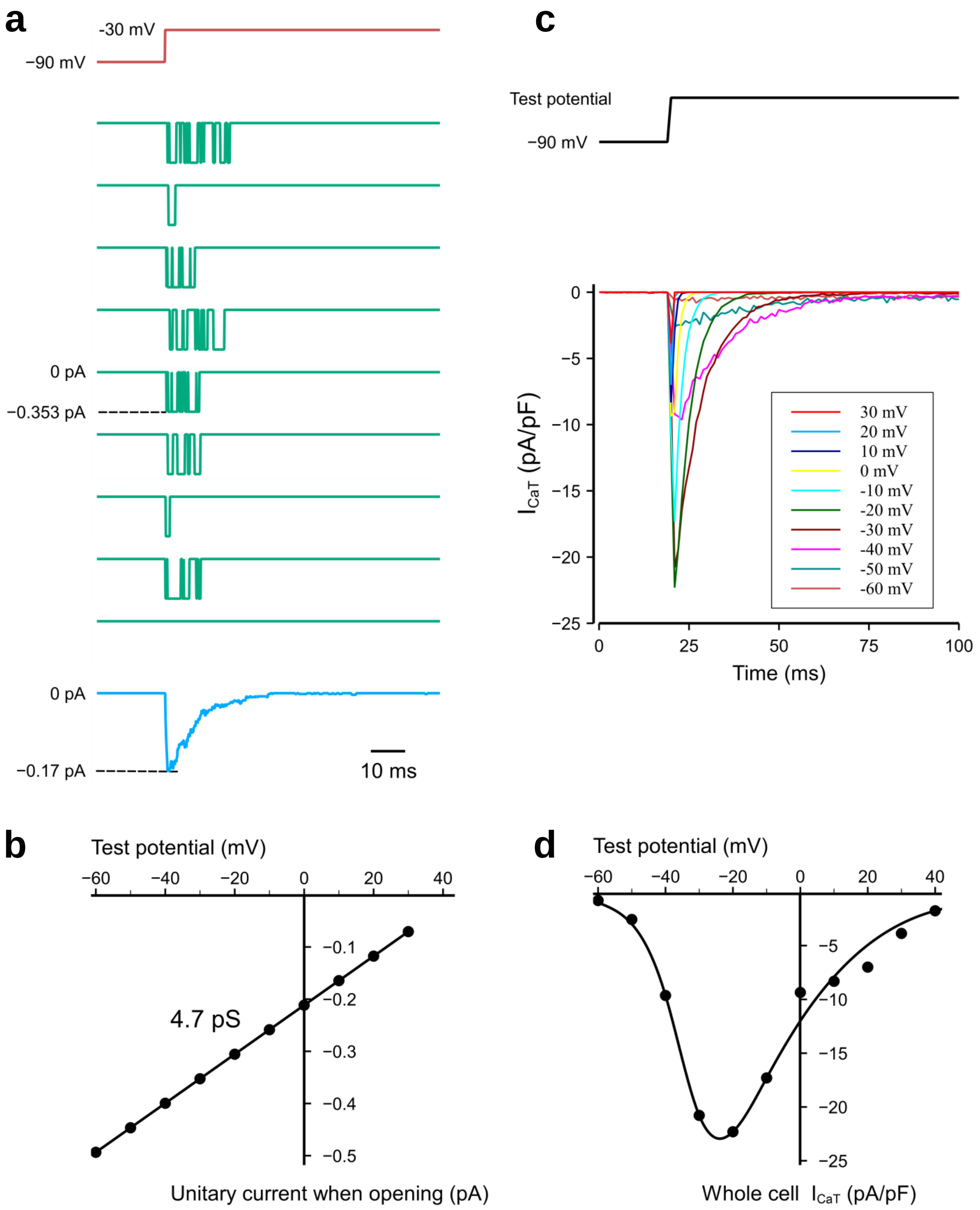

Supplementary Figure 10: Unitary and whole cell currents through the T-type Ca^{2+} channel. **a.** Voltage clamp protocol (red), unitary current traces (green; 9 consecutive sweeps shown) and ensemble average current from 200 consecutive sweeps (blue). The channel was held at -90 mV for 500 ms and then tested at -30 mV for 200 ms. **b.** Current-voltage relationship of the unitary T-type Ca^{2+} channel. Solid circles show the maximum current when the channel was open during 10 sweeps. The slope of the line indicating the unitary channel conductance was ~4.7 pS. c**.** Whole cell current from 2,970 unitary T-type Ca^{2+} channels with a holding potential of -90 mV and test potentials from -60 to +30 mV. **d.** Current-voltage relationship of the whole cell T-type Ca^{2+} current.

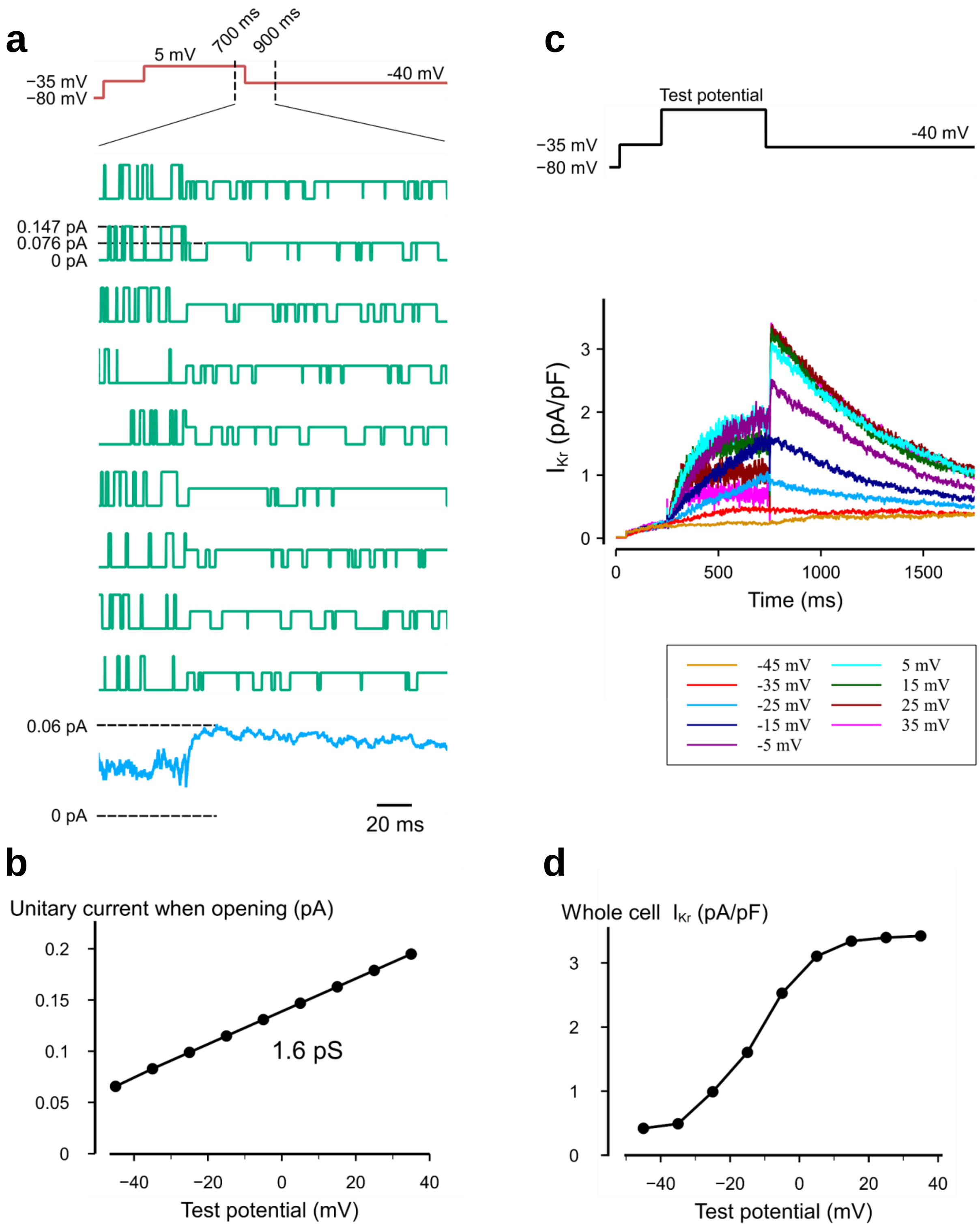

Supplementary Figure 11: Unitary and whole cell currents through the ERG channel. a. Voltage clamp protocol (red), unitary current traces (green; 9 consecutive sweeps shown) and ensemble average current from 200 consecutive sweeps (blue). The channel was held at -80 mV for 500 ms preceding the voltage clamp protocol. Current traces shown are from the time window 700 - 900 ms. **b.** Current-voltage relationship of the unitary ERG channel. Solid circles show the maximum current when the channel was open on the return to -40 mV during 10 sweeps. The slope of the line indicating the unitary channel conductance was 1.6 pS. **c.** Whole cell current from 2,610 unitary channels with the shown protocol. **d.** Current-voltage relationship of the whole cell ERG current.

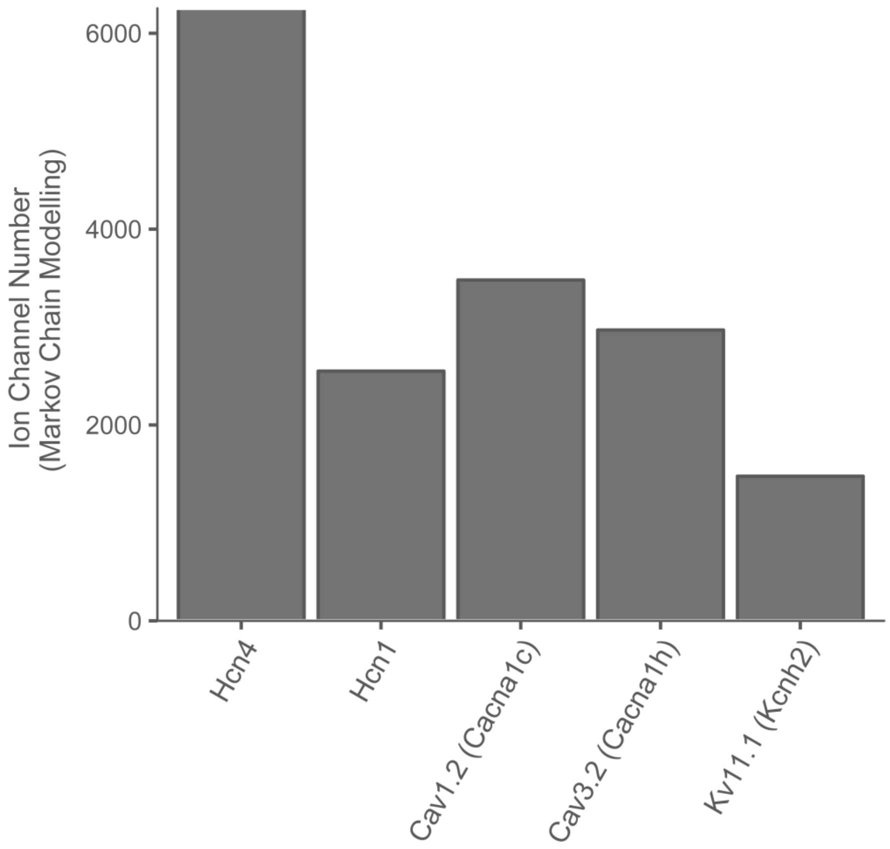

Supplementary Figure 12: Summary of ion channel copy numbers estimated from the Markov chain models presented in Figure 5 and Supplementary Figures 5-8.

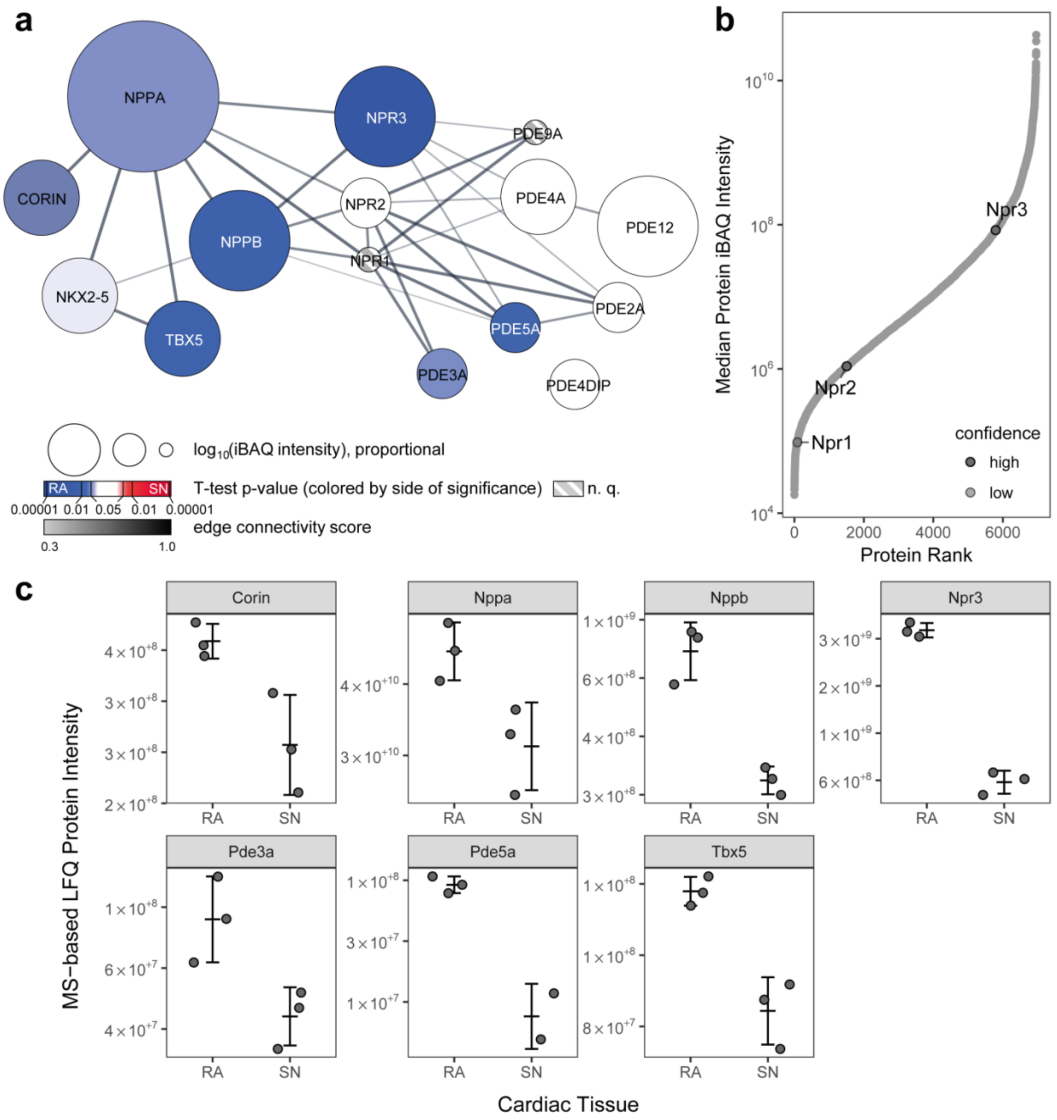

Supplementary Figure 13: Proteins involved in natriuretic peptide signalling are differentially expressed between sinus node (SN) and right atrium (RA). a. Protein association network of atrial natriuretic peptide (NPPA or ANP) retrieved from STRING [57] shows higher expression in right atria for eight proteins. Nodes are colored by significance (red – significantly higher abundant in SN, blue – significantly higher abundant in RA) and node size represents relative protein abundance. **b.** Rank plot showing all proteins in the dataset ranked from lowest to highest intensity. Npr3 was the most and Npr1 the least abundant of natriuretic peptide receptorss **c.** Protein expression in SN and RA for significantly differentially expressed proteins in the network (Student's t-test p-value < 0.05), LFQ: label-free quantification. Error bars represent mean ± standard deviation. Source data are provided as a Source Data file.

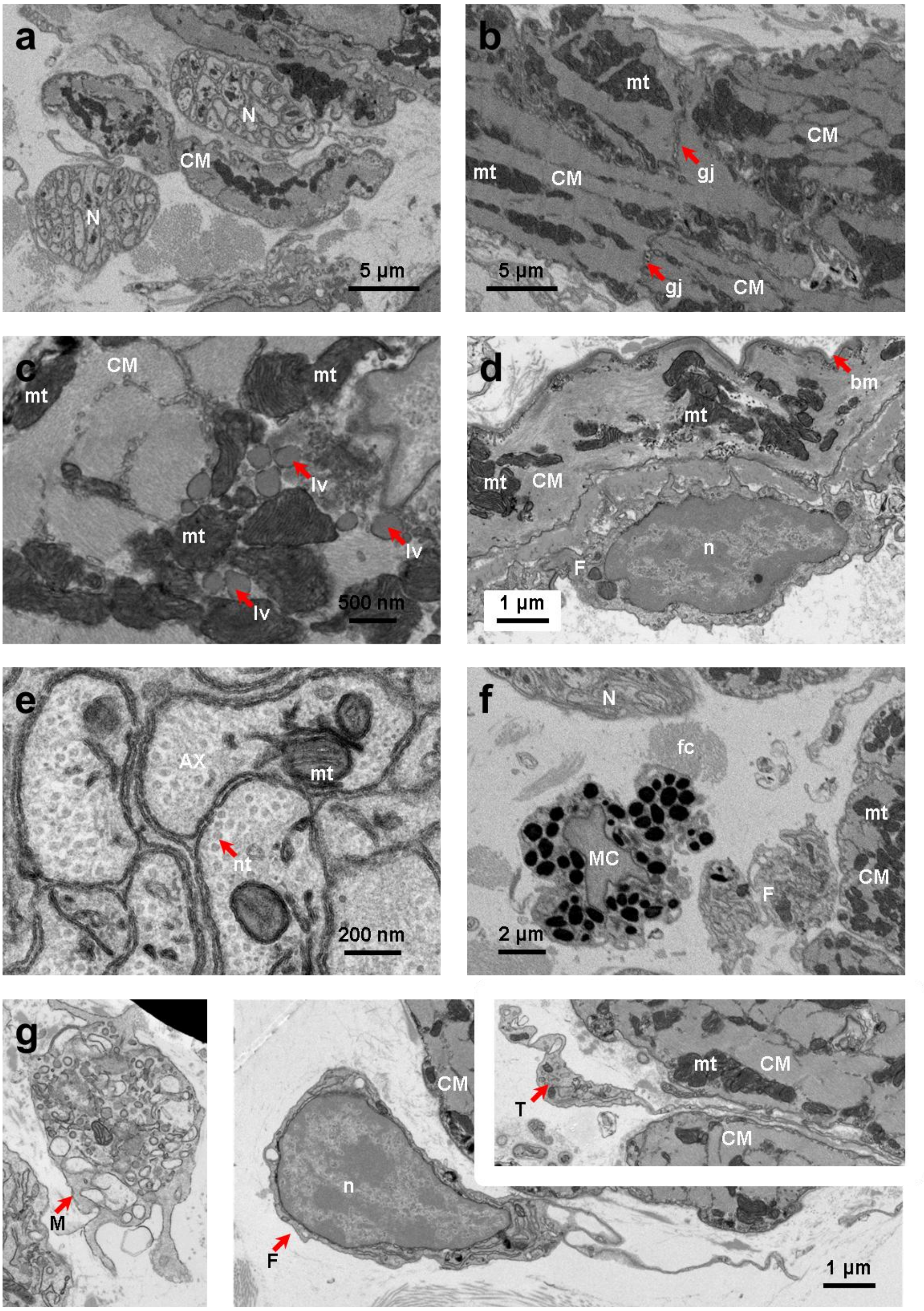

Supplementary Figure 14: Cellular heterogeneity of mouse sinus node as seen by transmission electron microscopy: a. A loosely spread out sinus node cardiomyocyte (CM) with dense myofibrils oriented in different directions located nearby two unmyelinated nerve endings (N). **b**. gap junctions (gj) interconnecting CMs in sinus node. Mitochondria (mt) are labelled. **c**. high magnification view showing lipid vescicles (lv) inside a sinus node CM. **d**. longitudinal section of a sinus node CM showing basement membrane (bm). An adjoining fibroblast (F) with a large nucleus (n) can be seen. **e**. cross section of nerve showing individual axons (AX), neurotubules (nt) and mitochondria (mt). **f-g**. typical mast cell (MC), macrophage (M), fibroblast (F) and telocyte (T) in mouse sinus node. Scale bars are shown for individual images.

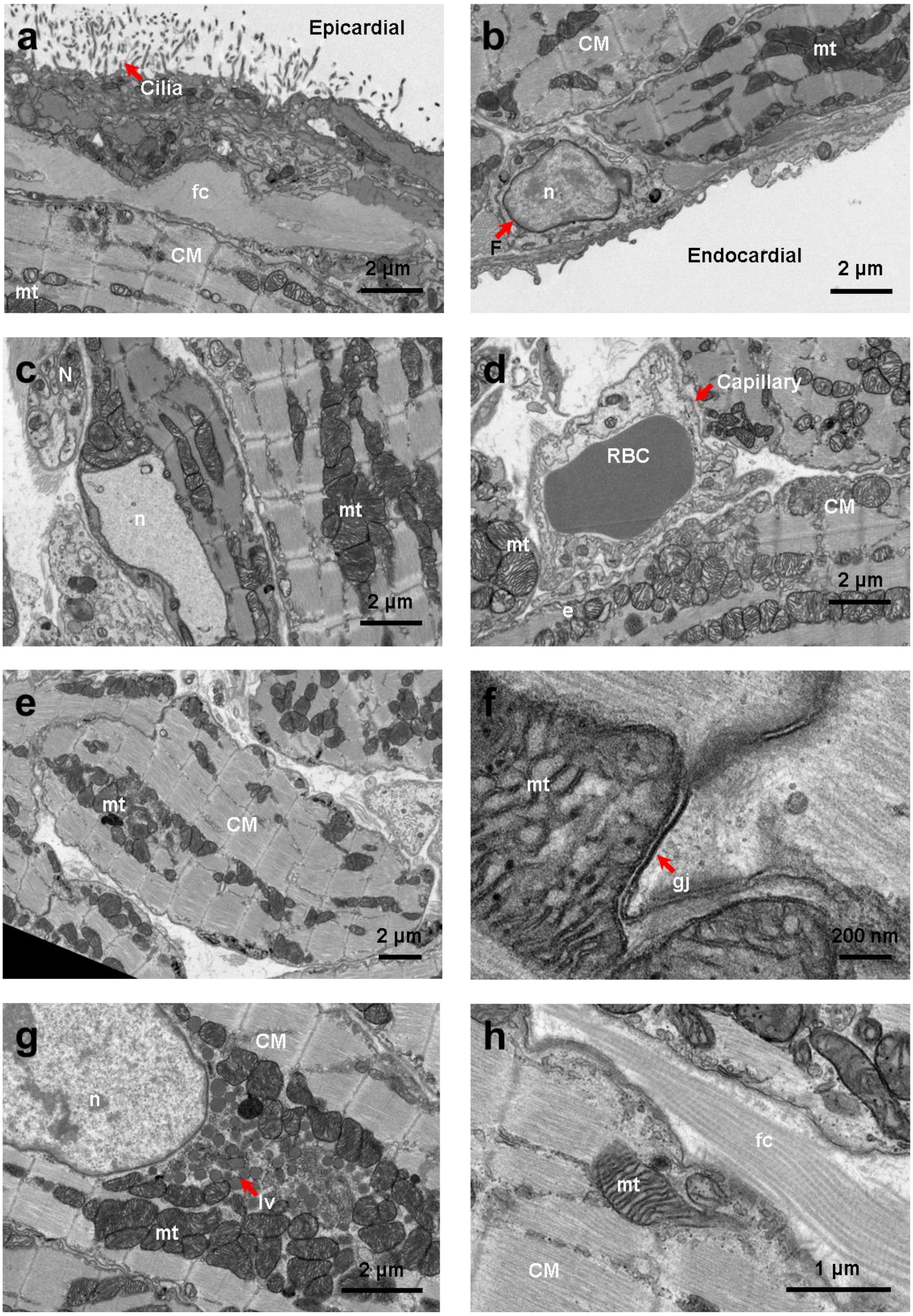

Supplementary Figure 15: Transmission electron micrographs of right atrial tissue in mouse: a. Right atrial epicardial end with cilia, layer of fibrillar collagen (fc) and cardiomyocyte (CM) with regularly arranged dense myofibrils oriented along the length of the cell. Mitochondria (mt) are labelled. **b.** Endocardial surface showing a fibroblast (F) with prominent nucleus (n) and tightly arranged cardiomyocytes (CM) with regular myofibrils and mitochondria (mt). **c.** unmyelinated nerve fiber (N) in close proximity to cardiomyocyte (CM) with prominent nucleus (n) and mitochondria (mt). **d.** Capillary with intact red blood cell (RBC). **e.** A tightly packed right atrial cardiomyocyte (CM) with dense myofibrils oriented along the length of the cell. **f.** gap junctions (gj) interconnecting right atrial cardiomyocytes (CM). **g.** intracellular lipid vesicles (lv) encircled by mitochondria (mt) and nucleus (n). **h.** intercellular fibrillar collagen (fc). Scale bars in each panel denote distance in µm.

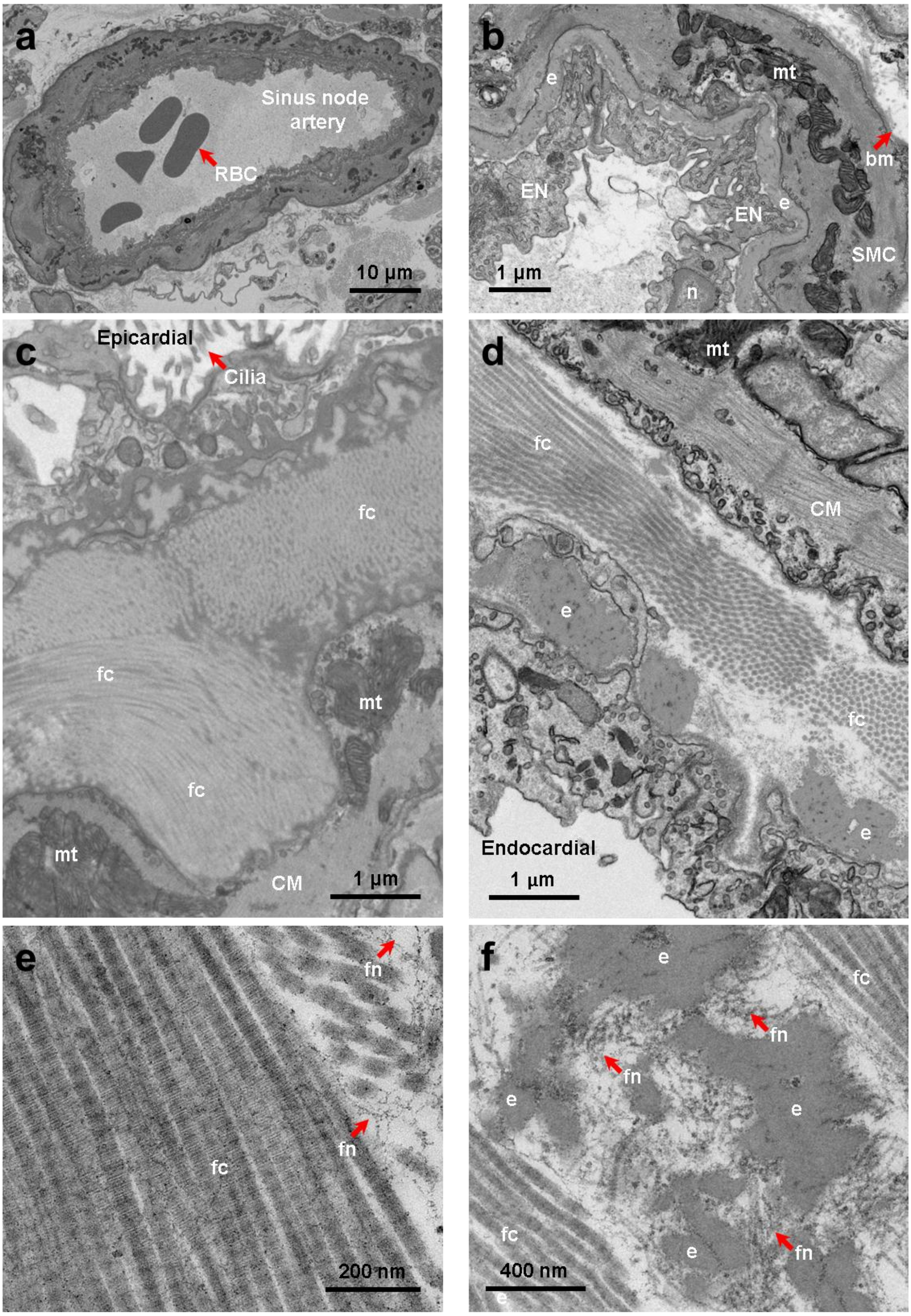

Supplementary Figure 16: Transmission electron micrographs of sinus node artery and extracellular matrix components in mouse sinus node: **a.** cross sectional view of mouse sinus node artery with intact red blood cells (RBCs). **b.** High magnification micrograph of sinus node artery showing an internal endothelial cell (EN) layer, elastic lamillae (e) and external smooth muscle cell (SMC). Bm, basement membrane; mt, mitochondria; n, nucleus. **c.** cilia and tightly packed collagen fibrils (fc) on the epicardial surface of sinus node. CM, cardiomyocyte; mt, mitochondria. **d.** fibrillary collagen (fc) and elastin (e) on endocardial surface. **e-f.** high resolution images of fibrillar collagen (fc), fibronectin (fn) and elastin (e). Scale bars are shown for individual images.

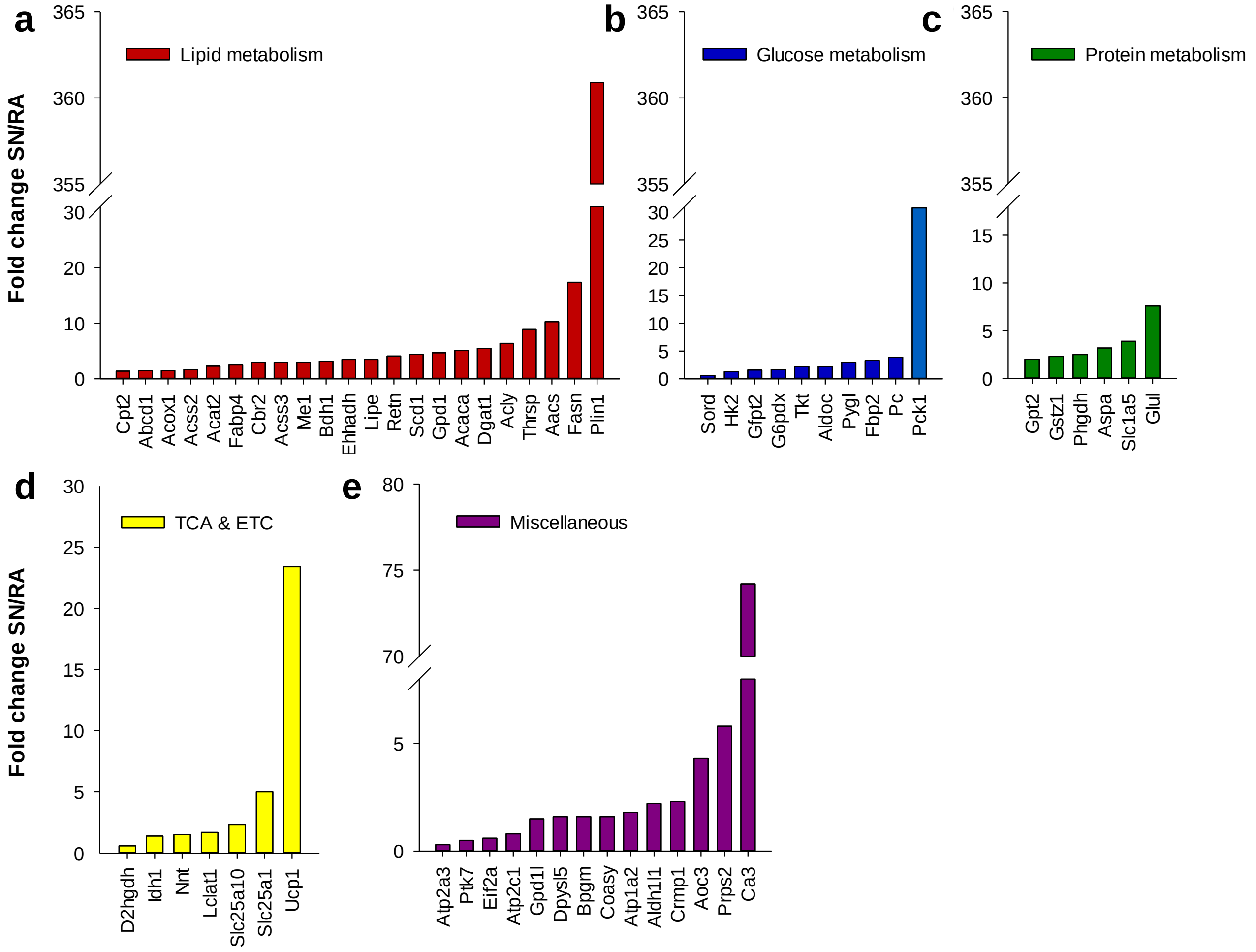

Supplementary Figure 17: Fold-change in expression of metabolic proteins between sinus node and right atria. Fold-changes are based on Volcano Plot analysis (see also Figure 6 and Supplementary Table 2) and shown for key proteins involved in **a.** lipid metabolism, **b.** glucose metabolism, **c.** protein metabolism, **d.** tricarboxylic acid (TCA) cycle and electron transport chain (TCA), **e.** miscellaneous others.

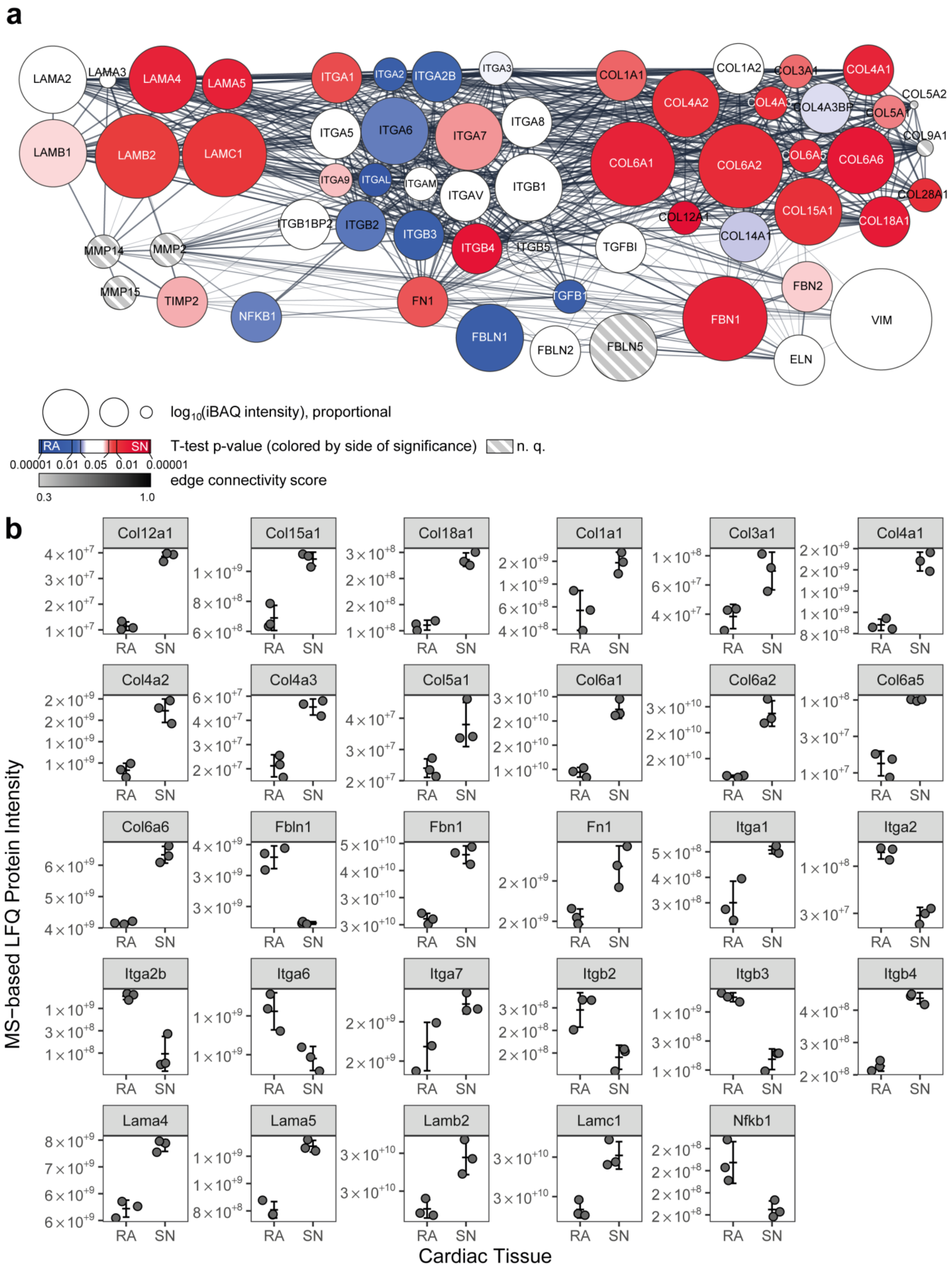

Supplementary Figure 18: Extracellular matrix (ECM) proteins are differentially expressed between sinus node (SN) and right atrium (RA). a. Protein association network of ECM proteins retrieved from STRING [57]. Nodes are colored by significance (red – significantly higher abundant in SN, blue – significantly higher abundant in RA) and node size represents relative protein abundance. **b.** Protein expression in SN and RA for significantly differentially expressed ECM proteins (Student's t-test p-value < 0.05), LFQ: label-free quantification. Error bars represent mean ± standard deviation. Source data are provided as a Source Data file.

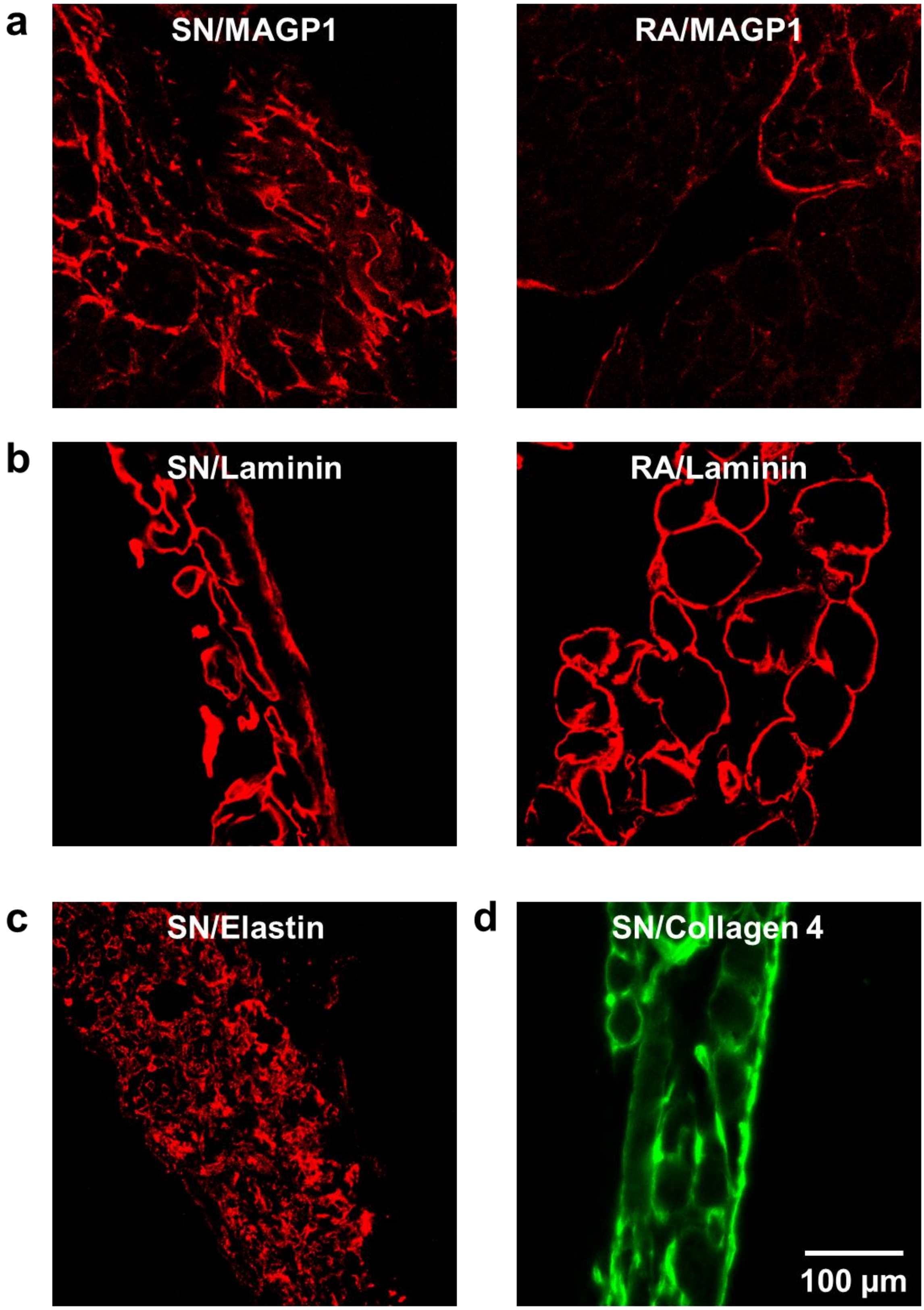

Supplementary Figure 19: Immunohistochemical staining of proteins in sinus node and atrial tissue sections from mice. a. MAGP1 (labels collagen VI), **b.** laminin (basement membrane marker), **c.** elastin and **d.** collagen IV. Representative scale bar is shown in panel d and denotes 100 µm.

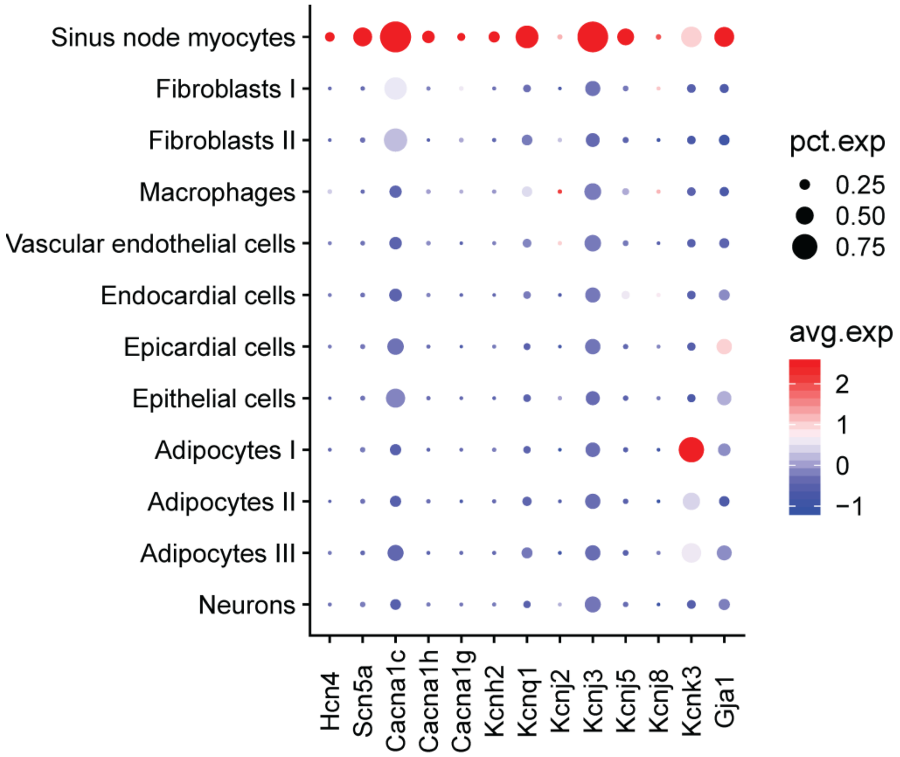

Supplementary Figure 20: Dot plot demonstrating the centered and scaled expression level of membrane clock ion channels across cell types illustrates that they are predominantly expressed in the sinus node myocytes. t-SNE, t-Distributed Stochastic Neighbor Embedding.

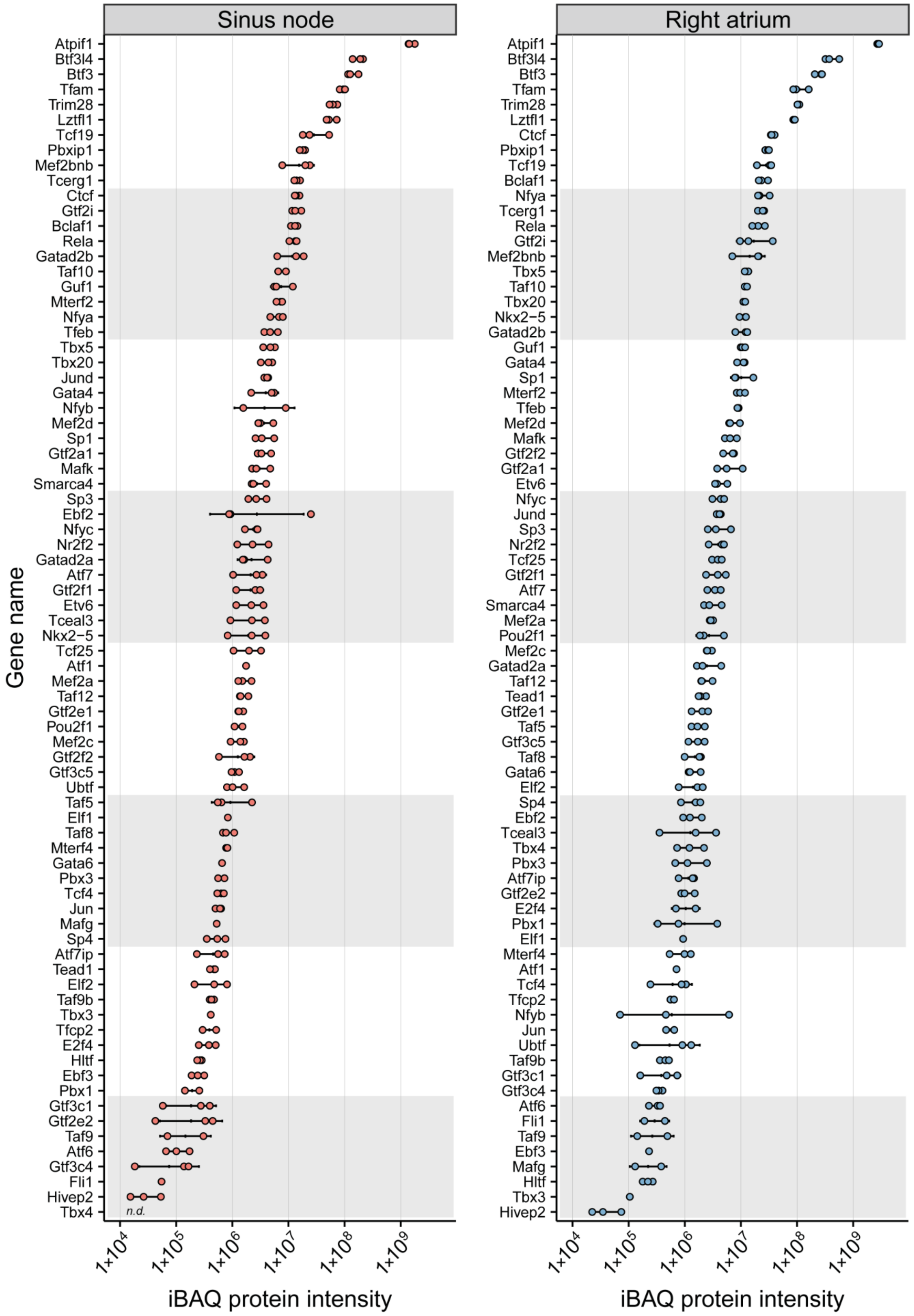

Supplementary Figure 21: Transcription factors expressed in Sinus node (SN) and right atrium (RA). Intensity-based absolute quantification (iBAQ) of 78 identified transcription factors spanning five orders of magnitude. Proteins are ranked from highest to lowest abundance in each chamber. Error bars represent mean +/- standard deviation. Source data are provided as a Source Data file.

SUPPLEMENTARY REFERENCES

1. Tellez, J. O. et al. Differential expression of ion channel transcripts in atrial muscle and sinoatrial node in rabbit. Circulation Research 99, 1384-1393 (2006).
2. Lei, M., Zhang, H., Grace, A. A. & Huang, C. L. SCN5A and sinoatrial node pacemaker function. Cardiovasc Res 74, 356-65 (2007).
3. Marionneau, C. et al. Specific pattern of ionic channel gene expression associated with pacemaker activity in the mouse heart. The Journal of Physiology 562, 223-234 (2005).
4. Roden, D. M., Balser, J. R., George, A. L., Jr. & Anderson, M. E. Cardiac ion channels. Annu.Rev.Physiol 64, 431-475 (2002).
5. Nerbonne, J. M., Nichols, C. G., Schwarz, T. L. & Escande, D. Genetic manipulation of cardiac K + channel function in mice: what have we learned, and where do we go from here? Circulation Research 89, 944-956 (2001).
6. Yanni, J. et al. Changes in ion channel gene expression underlying heart failure-induced sinoatrial node dysfunction. Circulation: Heart Failure 4, 496-508 (2011).
7. Mesirca, P., Torrente, A. G. & Mangoni, M. E. Functional role of voltage gated Ca(2+) channels in heart automaticity. Front Physiol 6, 19 (2015).
8. Grant, A. O. Cardiac ion channels. Circ Arrhythm Electrophysiol 2, 185-94 (2009).
9. Cordeiro, J. M. et al. Regional variation of the inwardly rectifying potassium current in the canine heart and the contributions to differences in action potential repolarization. J Mol Cell Cardiol 84, 52-60 (2015).
10. Dobrzynski, H. et al. Structure, function and clinical relevance of the cardiac conduction system, including the atrioventricular ring and outflow tract tissues. Pharmacology & Therapeutics 139, 260-288 (2013).
11. Cha, T. J. et al. Kir3-based inward rectifier potassium current: potential role in atrial tachycardia remodeling effects on atrial repolarization and arrhythmias. Circulation 113, 1730-7 (2006).
12. Sanchez-Rodriguez, I. et al. Activation of G-protein-gated inwardly rectifying potassium (Kir3/GirK) channels rescues hippocampal functions in a mouse model of early amyloid-beta pathology. Sci Rep 7, 14658 (2017).
13. Putzke, C. et al. The acid-sensitive potassium channel TASK-1 in rat cardiac muscle. Cardiovasc Res 75, 59-68 (2007).
14. Staudacher, K. et al. Carvedilol targets human K2P 3.1 (TASK1) K+ leak channels. Br J Pharmacol 163, 1099-110 (2011).
15. Gurney, A. M. et al. Two-pore domain K channel, TASK-1, in pulmonary artery smooth muscle cells. Circ Res 93, 957-64 (2003).
16. Bers, D. M. & Stiffel, V. M. Ratio of ryanodine to dihydropyridine receptors in cardiac and skeletal muscle and implications for E-C coupling. Am J Physiol 264, C1587-93 (1993).
17. Takeshima, H., Venturi, E. & Sitsapesan, R. New and notable ion-channels in the sarcoplasmic/endoplasmic reticulum: do they support the process of intracellular Ca(2)(+) release? J Physiol 593, 3241-51 (2015).
18. Kurabayashi, M., Komuro, I., Tsuchimochi, H., Takaku, F. & Yazaki, Y. Molecular cloning and characterization of human atrial and ventricular myosin alkali light chain cDNA clones. The Journal of biological chemistry 263, 13930-6 (1988).
19. Barth, A. S. et al. Functional profiling of human atrial and ventricular gene expression. Pfluegers Archiv - European Journal of Physiology 450, 201-208 (2005).
20. Tabibiazar, R., Wagner, R. a., Liao, A. & Quertermous, T. Transcriptional Profiling of the Heart Reveals Chamber-Specific Gene Expression Patterns. Circulation Research 93, 1193-1201 (2003).
21. Lu, Z. Q., Sinha, A., Sharma, P., Kislinger, T. & Gramolini, A. O. Proteomic analysis of human fetal atria and ventricle. Journal of Proteome Research 13, 5869-78 (2014).

22. Boyett, M. R., Honjo, H. & Kodama, I. The sinoatrial node, a heterogeneous pacemaker structure. Cardiovasc Res 47, 658-87 (2000).
23. Reiser, P. J., Portman, M. A., Ning, X. H. & Schomisch Moravec, C. Human cardiac myosin heavy chain isoforms in fetal and failing adult atria and ventricles. Am J Physiol Heart Circ Physiol 280, H1814-20 (2001).
24. Wessels, A., Vermeulen, J. L. M., Viragh, S. & Moorman, A. F. M. The Ontogenesis of Myosin Heavy-Chain Isoforms in the Developing Human Heart. Ann Ny Acad Sci 588, 461-464 (1990).
25. Ishikawa, T. et al. Novel Mutation in the alpha-Myosin Heavy Chain Gene Is Associated With Sick Sinus Syndrome. Circulation-Arrhythmia and Electrophysiology 8, 400-U200 (2015).
26. Peng, W. H. et al. Dysfunction of Myosin Light-Chain 4 (MYL4) Leads to Heritable Atrial Cardiomyopathy With Electrical, Contractile, and Structural Components: Evidence From Genetically-Engineered Rats. Journal of the American Heart Association 6 (2017).
27. Liu, J., Dobrzynski, H., Yanni, J., Boyett, M. R. & Lei, M. Organisation of the mouse sinoatrial node: structure and expression of HCN channels. Cardiovascular Research 73, 729-738 (2007).
28. Egom, E. E. et al. Impaired sinoatrial node function and increased susceptibility to atrial fibrillation in mice lacking natriuretic peptide receptor C. J Physiol 593, 1127-46 (2015).
29. van Weerd, J. H. & Christoffels, V. M. The formation and function of the cardiac conduction system. Development 143, 197-210 (2016).
30. Mardinoglu, A. et al. Defining the human adipose tissue proteome to reveal metabolic alterations in obesity. J Proteome Res 13, 5106-19 (2014).
31. Ahmadian, M. et al. PPARγ signaling and metabolism: the good, the bad and the future. Nature Medicine 19, 557 (2013).
32. Doll, S. et al. Region and cell-type resolved quantitative proteomic map of the human heart. Nature Communications 8, 1469 (2017).
33. Lindskog, C. et al. The human cardiac and skeletal muscle proteomes defined by transcriptomics and antibody-based profiling. BMC Genomics 16, 475 (2015).
34. Dobrzyn, P. et al. Expression of lipogenic genes is upregulated in the heart with exercise training-induced but not pressure overload-induced left ventricular hypertrophy. American Journal of Physiology-Endocrinology and Metabolism 304, E1348-E1358 (2013).
35. AbdAlla, S. et al. Up-Regulation of the Cardiac Lipid Metabolism at the Onset of Heart Failure. Cardiovascular & Hematological Agents in Medicinal Chemistry 9, 190-206 (2011).
36. Matsui, H. et al. Stearoyl-CoA desaturase-1 (SCD1) augments saturated fatty acid-induced lipid accumulation and inhibits apoptosis in cardiac myocytes. PLoS ONE 7, e33283 (2012).
37. Razani, B. et al. Fatty Acid Synthase Modulates Homeostatic Responses to Myocardial Stress. Journal of Biological Chemistry 286, 30949-30961 (2011).
38. Li, N. et al. Molecular Mapping of Sinoatrial Node HCN Channel Expression in the Human Heart. Circulation: Arrhythmia and Electrophysiology 8, 1219-1227 (2015).
39. Shirani, J., Berezowski, K. & Roberts, W. C. Quantitative measurement of normal and excessive (cor adiposum) subepicardial adipose tissue, its clinical significance, and its effect on electrocardiographic QRS voltage. The American Journal of Cardiology 76, 414-418 (1995).
40. Wu, Y., Zhang, A., Hamilton, D. J. & Deng, T. Epicardial Fat in the Maintenance of Cardiovascular Health. Methodist DeBakey Cardiovascular Journal 13, 20-24 (2017).
41. Aldiss, P. et al. 'Browning' the cardiac and peri-vascular adipose tissues to modulate cardiovascular risk. International Journal of Cardiology 228, 265-274 (2017).
42. Sacks, H. S. et al. Uncoupling Protein-1 and Related Messenger Ribonucleic Acids in Human Epicardial and Other Adipose Tissues: Epicardial Fat Functioning as Brown Fat. Molecular Endocrinology 23, 1519-1520 (2009).
43. Sacks, H. & Symonds, M. E. Anatomical locations of human brown adipose tissue: functional relevance and implications in obesity and type 2 diabetes. Diabetes 62, 1783-90 (2013).
44. Matloch, Z., Kotulak, T. & Haluzik, M. The role of epicardial adipose tissue in heart disease. Physiol Res 65, 23-32 (2016).
45. Roe, N. D., Handzlik, M. K., Li, T. & Tian, R. The Role of Diacylglycerol Acyltransferase (DGAT) 1 and 2 in Cardiac Metabolism and Function. Scientific Reports 8, 4983 (2018).

46. Heier, C. & Haemmerle, G. Fat in the heart: The enzymatic machinery regulating cardiac triacylglycerol metabolism. Biochimica et Biophysica Acta (BBA) - Molecular and Cell Biology of Lipids 1861, 1500-1512 (2016).
47. Welte, M. A. Expanding roles for lipid droplets. Curr Biol 25, R470-81 (2015).
48. Wang, H., Lei, M., Hsia, R.-c. & Sztalryd, C. in Methods in Cell Biology (eds. Yang, H. & Li, P.) 129-149 (Academic Press, 2013).
49. Goldberg, I. J., Trent, C. M. & Schulze, P. C. Lipid Metabolism and Toxicity in the Heart. Cell Metabolism 15, 805-812 (2012).
50. Puchalska, P. & Crawford, P. A. Multi-dimensional Roles of Ketone Bodies in Fuel Metabolism, Signaling, and Therapeutics. Cell Metabolism 25, 262-284 (2017).
51. Newman, J. C. & Verdin, E. Ketone bodies as signaling metabolites. Trends in Endocrinology & Metabolism 25, 42-52 (2014).
52. Cotter, D. G., Schugar, R. C. & Crawford, P. A. Ketone body metabolism and cardiovascular disease. American Journal of Physiology-Heart and Circulatory Physiology 304, H1060-H1076 (2013).
53. Verkerk, A. O. et al. Pacemaker current (If) in the human sinoatrial node. European Heart Journal 28, 2472-2478 (2007).
54. Chandler, N. J. et al. Molecular architecture of the human sinus node - insights into the function of the cardiac pacemaker. Circulation 119, 1562-1575 (2009).
55. Tellez, J. O. et al. Differential expression of ion channel transcripts in atrial muscle and sinoatrial node in rabbit. Circulation Research 99, 1384-93 (2006).
56. Vedantham, V., Galang, G., Evangelista, M., Deo, R. C. & Srivastava, D. RNA sequencing of mouse sinoatrial node reveals an upstream regulatory role for Islet-1 in cardiac pacemaker cells. Circulation research 116, 797-803 (2015).
57. Szklarczyk, D. et al. The STRING database in 2017: quality-controlled protein–protein association networks, made broadly accessible. Nucleic Acids Research 45, D362-D368 (2017).
