# Supplementary material for: Quantitative proteomics and single-nucleus transcriptomics of the sinus node elucidates the foundation of cardiac pacemaking
Source: Nat Commun. 2019 Jun 28;10:2889. doi: 10.1038/s41467-019-10709-9 (PMC6599035; doi:10.1038/s41467-019-10709-9)
Supplement: Supplementary file 3 — Description of Additional Supplementary Files [file 41467_2019_10709_MOESM3_ESM.docx]

**Description of Supplementary Files**

**File Name:** **Supplementary Data 1.**

**Description:** *All Proteins identified in sinus node and right atrial tissue*. Proteomic investigation of sinus nodal and adjacent right atrial tissue in mice resulted in identification of 7226 proteins.

**File Name:** **Supplementary Data 2.**

**Description:** *All ion channels identified in sinus node and right atrial tissue*. Proteomic data on ion channels of major interest in sinus node and right atrium.

**File Name:** **Supplementary Data 3.**

**Description:** Ion channel copy number estimation in sinus node based on iBAQ intensities and arkov chain modeling.

**File Name:** **Supplementary Data 4.**

**Description:** *Significantly differentially expressed protein between sinus node and atrial muscle.* Out of 7,226 proteins identified, 5,511 could be quantified with high confidence at LFQ level (identified in all replicates in sinus node or atrial muscle). Volcano plot analysis resulted in identification of 575 significantly differentially expressed proteins at 5% false discovery rate, out of which 291 were more highly abundant in the sinus node, and 284 were more highly abundant in the atrial muscle.

**File Name:** **Supplementary Data 5.**

**Description:** *Global network analysis of significant differences between sinus node and atrial muscle.* A protein association network was retrieved from the STRING database for all proteins significantly different between sinus node and atrial muscle (from Volcano Plot analysis). The network was clustered and enrichment of gene ontology terms was calculated for single clusters or tightly connected clusters. The table contains a simplified summary of the enrichment analysis as visualised in the network.

**File Name:** **Supplementary Data 6.**

**Description:** *Mean RNA transcript count in twelve cell clusters identified in sinus node tissue.* Single-nuclei RNA transcriptomic sequencing of sinus nodal tissue resulted in the measurement of 27998 genes in 5357 nuclei. Modularity-based clustering through the smart local moving (SLM) algorithm was used to identify twelve subpopulations which were manually annotated using the marker genes shown in Figure 9b. The table contains the average number of transcripts per gene within each cell cluster.

**File Name:** **Supplementary Data 7.**

**Description:** *All statistically significantly differentially expressed transcripts*. Differential expression analysis was performed between each cluster and all other cells with the MAST method from the MAST R package. The table contains pvalues, fold-changes, the proportions of cells inside and outside the cluster expressing the gene, and p-values adjusted for multiple testing using the Bonferroni method based on the total number of genes in the dataset.

**File Name:** **Supplementary Movie 1.**

**Description:** Serial transmission electron micrograph slices of the mouse sinus node illustrated in Fig. 8a are compiled and shown in this video. Sinus node myocytes are loosely arranged in extensive extracellular matrix. Also note the abundance of cilia on the epicardial surface. Scale bar denotes 10μm.

**File Name: Supplementary Movie 2.**

**Description:** Serial transmission electron micrograph slices of the epicardial end of mouse atrial tissue are compiled in this video. Atrial cardiomyocytes are tightly arranged and show well developed contractile filaments. Scale bar denotes 10μm.
