# Supplementary material for: Quantitative proteomics and single-nucleus transcriptomics of the sinus node elucidates the foundation of cardiac pacemaking
Source: Nat Commun. 2019 Jun 28;10:2889. doi: 10.1038/s41467-019-10709-9 (PMC6599035; doi:10.1038/s41467-019-10709-9)
Supplement: Supplementary file 13 — Reporting Summary [file 41467_2019_10709_MOESM13_ESM.pdf]

nature research

Corresponding author(s): Alicia Lundby

Reporting Summary

Nature Research wishes to improve the reproducibility of the work that we publish. This form provides structure for consistency and transparency in reporting. For further information on Nature Research policies, see Authors & Referees and the Editorial Policy Checklist.

Statistical parameters

When statistical analyses are reported, confirm that the following items are present in the relevant location (e.g. figure legend, table legend, main text, or Methods section).

n/a	Confirmed	
☐	☒	The exact sample size (*n*) for each experimental group/condition, given as a discrete number and unit of measurement
☐	☒	An indication of whether measurements were taken from distinct samples or whether the same sample was measured repeatedly
☐	☒	The statistical test(s) used AND whether they are one- or two-sided<br>*Only common tests should be described solely by name; describe more complex techniques in the Methods section.*
☐	☒	A description of all covariates tested
☐	☒	A description of any assumptions or corrections, such as tests of normality and adjustment for multiple comparisons
☐	☒	A full description of the statistics including central tendency (e.g. means) or other basic estimates (e.g. regression coefficient) AND variation (e.g. standard deviation) or associated estimates of uncertainty (e.g. confidence intervals)
☐	☒	For null hypothesis testing, the test statistic (e.g. *F*, *t*, *r*) with confidence intervals, effect sizes, degrees of freedom and *P* value noted<br>*Give P values as exact values whenever suitable.*
☒	☐	For Bayesian analysis, information on the choice of priors and Markov chain Monte Carlo settings
☒	☐	For hierarchical and complex designs, identification of the appropriate level for tests and full reporting of outcomes
☐	☒	Estimates of effect sizes (e.g. Cohen's *d*, Pearson's *r*), indicating how they were calculated
☐	☒	Clearly defined error bars<br>*State explicitly what error bars represent (e.g. SD, SE, CI)*

Our web collection on statistics for biologists may be useful.

Software and code

Policy information about availability of computer code

Data collection	Xcalibur for LC-MS/MS. LSM5 Zeiss Pascal for Immuno. Gatan Microscopy Suite & IMOD for EM. Chromium system (10X genomics) and NovaSeq™ 6000 sequncing system (Illumina) for scRNAseq
Data analysis	MaxQuant, Perseus, Excel, R, ImageJ. For scRNAseq: Cellranger 3.0 pipeline (10x Genomics, USA) , R packages Seurat, DoubletFinder, and MAST

For manuscripts utilizing custom algorithms or software that are central to the research but not yet described in published literature, software must be made available to editors/reviewers upon request. We strongly encourage code deposition in a community repository (e.g. GitHub). See the Nature Research guidelines for submitting code & software for further information.

Data

Policy information about availability of data

All manuscripts must include a data availability statement. This statement should provide the following information, where applicable:

- Accession codes, unique identifiers, or web links for publicly available datasets
- A list of figures that have associated raw data
- A description of any restrictions on data availability

All mass spectrometry and RNA sequencing data is made available, details provided in manuscript.

Field-specific reporting

Please select the best fit for your research. If you are not sure, read the appropriate sections before making your selection.

☒ Life sciences ☐ Behavioural & social sciences ☐ Ecological, evolutionary & environmental sciences

For a reference copy of the document with all sections, see nature.com/authors/policies/ReportingSummary-flat.pdf

Life sciences study design

All studies must disclose on these points even when the disclosure is negative.

Sample size	Sample size was chosen based on collection of sufficient amount of tissue to perform mass spectrometry measurements on minimal number of animals.
Data exclusions	No data was excluded
Replication	LC-MS/MS measurements were performed in technical replicates, results were consistent.
Randomization	There was no randomization as both sets of biopsies (sinus node and atrial tissue) were in all instances collected from the same animal.
Blinding	Biopsies were collected by one person and samples were numbered. Subsequent sample preparation, measurement and data analysis was done by another person who did not know the identity of the samples.

Reporting for specific materials, systems and methods

Materials & experimental systems

n/a	Involved in the study
☒	☐ Unique biological materials
☐	☒ Antibodies
☒	☐ Eukaryotic cell lines
☒	☐ Palaeontology
☐	☒ Animals and other organisms
☒	☐ Human research participants

Methods

n/a	Involved in the study
☒	☐ ChIP-seq
☒	☐ Flow cytometry
☒	☐ MRI-based neuroimaging

Antibodies

Antibodies used

- HCN4:
rabbit polyclonal anti-HCN4 (1:100; APC-052, Alomone Labs, Israel)
donkey anti-rabbit Fluorescein conjugate (1:100; AP182F, Millipore, USA) or
donkey anti-rabbit Cy3 conjugate (1:400; AP182C, Millipore, USA)
- Cx43:
rabbit polyclonal anti-Cx43 (1:1000; C6219, Sigma-Aldrich, UK)
donkey anti-rabbit, Fluorescein conjugate (1:100; AP182F, Millipore, USA)
- TASK-1:
rabbit polyclonal anti-KCNK3 (1:10; APC-024, Alomone Labs, Israel)
donkey anti-rabbit Cy3 conjugate (1:400; AP182C, Millipore, USA)
- MAGP-1:
goat polyclonal anti-MAGP-1 (1:200; sc-166075, Santa Cruz Biotechnology, USA)
donkey anti-goat Alexa Fluor 546 (1:400; A11056, Thermo Fisher Scientific, UK)
- Laminin:
rabbit polyclonal anti-laminin (1:200; L9393, Sigma-Aldrich, UK),
donkey anti-rabbit Cy3 conjugate (1:400; AP182C, Millipore, USA)
- Elastin:
rabbit polyclonal anti-elastin (1:100; ab21610, abcam, UK),
donkey anti-rabbit Cy3 conjugate (1:400; AP182C, Millipore, USA)
- Collagen IV:
rabbit polyclonal anti-collagen type IV (1:100; AB756P, Merck, UK)
goat anti-rabbit Alexa Fluor 488 (1:400; A11034, Thermo Fisher Scientific, UK)

Validation	All antibodies used were commercial antibodies validated by supplier.

Animals and other organisms

Policy information about studies involving animals; ARRIVE guidelines recommended for reporting animal research

Laboratory animals	C57BL/6J mice, 12-14 weeks old and 25-35 g body weight
Wild animals	Study did not involve wild animals
Field-collected samples	Study did not involve field-collected samples
